# Endophenotypes of Primary Osteoarthritis of the Hip Joint in the Bulgarian Population over 60 Years Old

**DOI:** 10.3390/life14050622

**Published:** 2024-05-11

**Authors:** Lyubomir Sapundzhiev, Tanya Sapundzhieva, Kamen Klinkanov, Martin Mitev, Kiril Simitchiev, Anastas Batalov

**Affiliations:** 1 Department of Propedeutics of Internal Diseases, Medical Faculty, Medical University of Plovdiv, 4001 Plovdiv, Bulgaria; taniasapundjieva@abv.bg (T.S.); kamenklinkanov@gmail.com (K.K.); abatalov@hotmail.com (A.B.); 2 Rheumatology Department, University Hospital ‘Pulmed’ Plovdiv, 4002 Plovdiv, Bulgaria; m.mitev97@gmail.com; 3 Department of Analytical Chemistry and Computer Chemistry, Faculty of Chemistry, University of Plovdiv, 4001 Plovdiv, Bulgaria; 4 Rheumatology Clinic, University Hospital ‘Kaspela’, 4000 Plovdiv, Bulgaria

**Keywords:** pHOA, endophenotypes, THR

## Abstract

**Aim.** To identify subgroups of patients with primary osteoarthritis of the hip joint (pHOA) with similar imaging and laboratory findings, disease evolution, and response to conventional therapies. **Methods.** We performed further statistical analyses on patient data from two published, double-blind, randomized, and placebo-controlled studies (DB-RCTs), which examined the effects of intra-articular corticosteroids (ia-CSs), hyaluronic acid (ia-HA)—KИ-109-3-0008/14.01.2014, and intravenous bisphosphonates (iv-BPs) -KИ- 109-3-0009/14.01.2014 compared to the country’s standard pHOA therapy. The data span an 8-year follow-up of 700 patients with pHOA, including: 1. Clinical parameters (WOMAC-A, B, C, and T; PtGA). 2. Laboratory markers (serum calcium and phosphate levels; 25-OH-D and PTH, markers for bone sCTX-I and cartilage uCTX-II turnover). 3. Radiological indicators: X-ray stage (Kellgren-Lawrence (K/L) and model (Bombelli/OOARSI), width (mJSW), speed (JSN mm/year), and zone of maximum narrowing of the joint space (max-JSN)—determining the type of femoral head migration (FHM). 4. DXA indicators: bone geometry (HAL; NSA; and MNW); changes in regional and total bone mineral density (TH-BMD, LS-BMD, and TB-BMD). 5. Therapeutic responses (OARSI/MCII; mJSW; JSNmm/yearly) to different drug regimens (iv-BP -zoledronic acid (ZA/-5 mg/yearly for 3 years)); ia-CS 40 mg methylprednisolone acetate, twice every 6 months; and ia-HA with intermediate molecular weight (20 mg/2 mL × 3 weekly applications, two courses every 6 months) were compared to standard of care therapy (Standard of Care/SC/), namely D3-supplementation according to serum levels (20–120 ng/mL; target level of 60 ng/mL), simple analgesics (paracetamol, up to 2.0 g/24 h), and physical exercises. The abovementioned data were integrated into a non-supervised hierarchical agglomerative clustering analysis (NHACA) using Ward’s linkage method and the squared Euclidean distance to identify different endophenotypes (EFs). Univariate and multivariate multinomial logistic regression analyses were performed to determine the impact of sex and FHM on clinical and radiographic regression of pHOA. **Results.** A baseline cluster analysis using incoming (M0) patient data identified three EFs: hypertrophic H-HOA, atrophic A-HOA, and intermediate I-HOA. These EFs had characteristics that were similar to those of patients grouped by radiographic stage and pattern (‘H’-RPs, ‘I’-RPs, and ‘A’-RPs), *p* < 0.05). The repeated cluster analysis of M36 data identified four EF pHOAs: 1. Hypertrophic (slow progressors, the influence of the type of femoral head migration (FHM) outweighing the influence of sex on progression), progressing to planned total hip replacement (THR) within 5 (K/LIII) to 10 (K/LII) years. 2. Intermediate (sex is more important than the FHM type for progression) with two subgroups: 2#: male-associated (slow progressors), THR within 4 (K/LIII) to 8 years. (K/LII). 2* Female-associated (rapid progressors), THR within 3 (K/LIII) to 5 (K/LII) years. 3. Atrophic (rapid progressors; the influence of FHM type outweighs that of sex), THR within 2 (K/LIII) to 4 (K/LII) years. Each EF, in addition to the patient’s individual progression rate, was also associated with a different response to the aforementioned therapies. **Conclusions.** Clinical endophenotyping provides guidance for a personalized approach in patients with pHOA, simultaneously assisting the creation of homogeneous patient groups necessary for conducting modern genetic and therapeutic scientific studies.

## 1. Introduction

**Figure 1:**
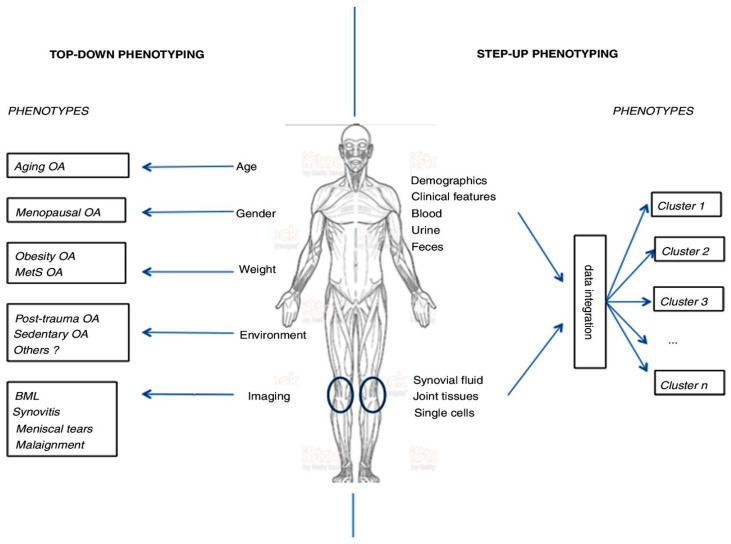
Endophenotypes (EF) definition according to Francis Berenbaum, ARD 2018 (with permission). On the left, ΕFs are defined based on the leading pathogenetic moment (the role of a confirmed risk factor), supported by imaging studies. On the right, EFs are defined through the integration and clustering of objective findings (demographics, clinical features, and laboratory data), realized with the help of statistical methods. The homogeneity of EF (non-dependent on the approach) is based on the number of individually collected data, and the quality of their consecutive integration. [6].

OA is a disease of synovial joints, engaging all the structures of these joints, such as the articular cartilage (AC), the subchondral bone (SB), the synovial tissue (ST), ligaments, the joint capsule, and periarticular muscles, resulting in an articular deficit [1]. The complex pathogenesis of OA consists of the interaction between various mechanical, inflammatory, and metabolic factors. Each of them is presented with a different grade of significance relevant to different localizations of the disease [1,2], as well as according to separate clinic-morphologic variants (endophenotypes) in one and the same localization [3]. These facts require the validation of different endophenotypes (EFs) of OA in their respective localizations [4,5,6] (Figure 1).

By definition [7], a phenotype refers to a complex of individual traits that are observable and measurable by simple methods. A person’s phenotype is determined by interactions between their genotype and environmental factors. In this context [3,4,5,6,7], EFs in different localizations of primary OA constitute subgroups of patients with similar demographics (ethnicity, gender, and age), anthropometrics (height and body mass index (BMI)), similar findings from imaging and laboratory studies, disease progression, and therapeutic responses to the present management.

Regarding OA of weight-bearing joints, unfortunately phenotyping and genotyping in primary knee OA (pKOA) is many years ahead compared to the phenotyping and genotyping of patients with primary HOA. This is probably due to the higher incidence of the disease, as well as its easier clinical and radiologic examinations and access to the knee joint for clinical interventions [8,9,10]. Analogies are also irrelevant in cases of hand osteoarthritis, where endophenotyping and genetic studies are performed “hand in hand” [11,12].

In primary HOA, attempts at endophenotyping begin with the introduction of the following three radiographic patterns (RPs): hypertrophic (‘H’-RP), atrophic (‘A’-RP), and intermediate or normotrophic (‘I’-RP) based on radiograph differences between them [13,14]. Among the respective RPs are established differences in radiograph progression [15,16,17,18,19,20,21], as well as differences in biomarkers of bone, cartilage, and synovial turnover [22,23,24,25], and in total and local bone mineral density (BMD) [26,27,28]. There are different therapeutic responses to different pharmacologic medications: ia-CS [29,30,31], ia-HA [31], NSAIDs, antibodies to neuronal growth factor [24,25], and oral and intravenous bisphosphonates [32,33,34]. At present, there are four established basic endophenotypes of primary HOA based upon RPs, BMD, and markers of bone, cartilage, and synovial turnover [22,23,24,25,35]: hypertrophic (‘H’-HOA), intermediate (‘I’-HOA), atrophic (‘A’-HOA), and rapidly progressive (‘RP’-HOA). 

Despite the methodology’s almost 50-year history and its published benefits [35], ΕF in primary HOA is still not recognized as a widely established standard in patient care and a base for scientific trials. The current study’s objectives are to identify the EFs of primary HOA in the Bulgarian patient population over 60 years of age and to establish this methodology as a component of the multifaceted care plan for these patients. 

## 2. Materials and Methods

We performed additional statistical processing of patient data from two published, double-blinded, randomized, and placebo-controlled studies (DB-RCTs), which tested the effect of ia-CS, ia-HA (KИ-109-3-0008/14.01.2014) [31], and iv-BP (ZA) [34] -KИ- 109-3-0009/14.01.2014 www.bda.bg. The results were compared with the country’s standard pHOA- D3 supplementation according to serum levels (20–120 ng/mL; target level of 60 ng/mL), simple analgesics (paracetamol, up to 2.0 g/24 h), and physical exercises. Both studies are part of a national program to actualize standards for the prevention, non-surgical treatment, and surgical treatment of OA, and they have the following characteristics in common:

1. *Inclusion criteria*: unilateral or bilateral primary HOA according to ACR criteria [36]; symptomatic criteria of WP ≥ 40/100 mm VAS [37], WOMAC-A ≥ 6/20, and WOMAC-C ≥ 30/68 [38]; and radiographic confirmation by the presence of II and III radiologic grades according to K/L classification [39].
2. *Exclusion criteria*: secondary HOA; significant malalignment (Valgus/Varus alignment ≥2°); US findings for hydrops/synovitis; clinical, laboratory, and radiograph findings of rapid progressive disease (RP-HOA) in atrophic models; intra-articular therapies or therapy with sulfate sugars, biocollagen, hyaluronic acid, diacerein, and avocado and soya extracts 6 months prior to the screening period; age below 60 and above 70 years; significant abnormalities in body mass index (/BMI/21 kg/m^2^ > BMI > 28 kg/m^2^); poor control of general diseases (arterial hypertension, diabetes mellitus, coronary and brain arteries disease, and thyroid dysfunction), affecting the duration of life expectancy and polypragmasy.
3. *Design*: strict adherence to OARSI recommendations for the design, planning, and trial protocol for patients with primary HOA [40]. Those included control and therapeutic groups of 25 patients with fixed sex distribution of females and males (15/10) in each group; six groups of patients presenting both radiograph grades (K/L-grade) and the three RP’s (K/L-II‘A’; K/L-II‘H’; K/L-II-‘I’; K/L-III‘A’; K/L-III‘H’; K/L-III‘I’); and DB-RCT and restricted block randomization.
4. *Indicators and follow-up periods*: 6-month intervals (clinical, US, and laboratory indicators) and 12-month intervals (X-Ray/DXA results); period span until an elective THR or informed consent withdrawal (3–10 years).

The observation on the effect of ia-CS/ia-HA in primary HOA [31] was carried out in three groups (total of 400 patients) with the following administrations:

✓ Standard of care (SC) + Methylprednisolone acetate–Depo Medrol^®^ 80 mg/2 mL. (Pfizer Inc. Distributed by Pharmacia & Upjohn Company LLC, division of Pfizer Inc., New York, NY, USA), two-time application—at months M0 and M6, n of patients = 150.
✓ SC + Euflexxa® -MMW-HA, 20 mg/2 mL, molecular weight 2.4–3.6 MD (Bio-Technology General -Israel Ltd./Be ‘er Tuvia, Kiryat Malachi, Israel/). Distributed by FERRING PHARMACEUTICALS INC./Parsippany, NJ, USA/-2 courses (M0/M6) of three consecutive weekly applications, n of patients = 150.
✓ SC + Placebo (Normal Saline, Pfizer Injectables, Sodium Chloride for Injection, 0.9%), a sterile solution packaged in a flip-top plastic vial of 10 mL × 25 per tray, manufactured by Pfizer Inc., and distributed by Pharmacia & Upjohn Company LLC, a division of Pfizer Inc. (New York, NY, USA). Placebo injections are intra-articular (2.0 mL), twice administered (Μ0/M6), n of patients = 100 (‘A’-RPs –excluded).

The observation on iv-BP [34] was done in two groups, including a total of 300 patients:

✓ SC + application of 5 mg. ZA (Aclasta^®^ 5 mg/100 mL Infusion, Novartis India Ltd., Mumbai, Maharashtra, India) in 3 consecutive years, n of patients = 150.
✓ SC + Placebo (Normal Saline, Pfizer Injectables, Sodium Chloride for Injection, 0.9% sterile solution packaged in a flip-top plastic vial 100 mL × 25 per tray, manufactured by Pfizer Inc. and distributed by Pharmacia & Upjohn Company LLC, a division of Pfizer Inc., New York, NY, USA). Placebo infusions contain 100 mL and are administered once in 3 consecutive years, with a total of 150 patients.

### 2.1. Specificities of the Studied Population

(a) *Incidence of different RPs*: The formation of a cohort of 700 randomized patients was preceded by a 12-month screening period in three centers (MHAT “Sofiamed”, Sofia, Bulgaria; MHAT “Pulmed”, Plovdiv, Bulgaria; and MHAT “Zdrave”, Pazardjik, Bulgaria), including a physical examination and X-rays of the hip joints of 2200 patients with primary HOA, of whom 1400 were female and 800 were male. This allowed us to determine the distribution of different RPs in the Bulgarian population over the age of sixty (Table 1).

The determined incidence, albeit close to the distribution pointed out by Ledingham et al. [15], based on large representative samples, showed a tendency towards a higher incidence of ‘A’-RPs among our population, while the incidence of ‘I’-RPs was lower. 

(b) *Vitamin D3 values and osteoporosis*: A population research on vitamin D3 (D3) levels in Bulgaria showed [41]:

✓ A severe deficit of D3 (<25 nmol/L/<10 ng/mL) in 15.1% of males, compared to 26.9% in females in the general population. In the age group over 60 years, the results showed a severe deficit in 20.2% of the males and in 28.2% of the females.
✓ A deficit of D3 (25 nmol/L < D3 < 49.9 nmol/L/10 ng/mL < D3 < 20 ng/mL) was established, respectively, in 52.3% of males and 55.3% of females.
✓ Normal values were observed in 19.4% of females and in 29.6% of males.

**Figure 2:**
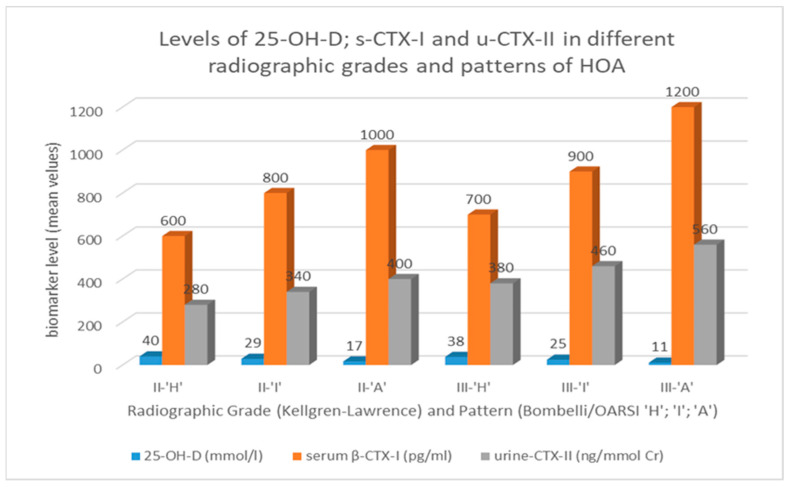
Values of 25-OH-D levels and levels of markers for bone (serum β-CTX-I) and cartilage (urine CTX-II) turnover in 700 patients (60–69 yrs.) at baseline (M0), according to radiographic grade and pattern: 25-OH-D –serum 25-hydroxy vitamin D levels in mmol/liter; serum β-CTX-I—beta-isomerized carboxy-terminal cross-linking telopeptide of type I collagen in picograms per milliliter; and urine CTX-II—C-terminal crosslinking telopeptides of collagen type II, presented as corrected concentrations of uCTX-II for urinary creatinine concentration, as ng/mmol Cr.

In our cohort of patients, a severe deficit of vitamin D3 was established in 21.4% (60/280) of males and in 29.5% (124/420) of females. A deficit of D3 was observed in 53.6% (150/280) of males and 56.2% (236/420) of females. Normal D3 levels were detected in 25% (70/280) of males and in 14.30% (60/420) of females, as all of them (100%) were associated with ‘H’-RPs, which is different from the deficit states associated with ‘A’-RPs/‘I’-RPs (Table 2 and Figure 2). Comparing our results to those of Borissova et al. [41], we should note the higher incidence of severe deficit and deficit of D3 in the patients in our cohort (mean 1.3%). This may be explained by the immobilization and the poor quality of life of patients with accompanying pHOA.

(c) *BMD population trials in Bulgaria among women* [42] *and men* [43]. For women above 50 years of age, the results showed osteoporosis in 16.8%, osteopenia in 45.5%, and normal BMD in 37.7% [42]. Among males over 60 years old, the reported incidence was osteoporosis in 14.1%, osteopenia in 42.8%, and normal BMD in 43.1% [43]. 

**Figure 3:**
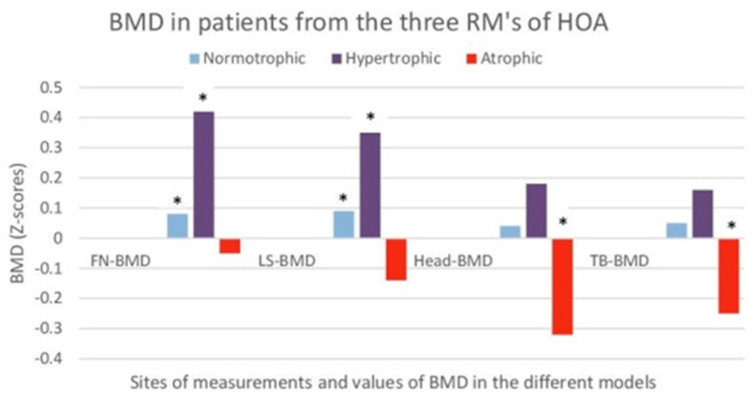
Bone mineral density (BMD) in 700 patients (60–69 years) of the three radiographic patterns (RMs) of hip osteoarthritis (atrophic, normotrophic, and hypertrophic). The results are shown as Z scores for FN-BMD (femoral neck), LS-BMD (lumbar spine), head-BMD (cranium), and TB-BMD (total body) for each radiographic pattern. Z-scores allowed comparisons with standardized (gender, age, and BMI) controls without hip osteoarthritis (HOA); *—a statistically significant difference (*p* < 0.05) in comparison with controls without HOA.

Among our cohort of patients, all of the ‘A’RPs presented lower BMD at all of the measured sites (TB-BMD, LS-BMD, and PF-BMD). Seventy percent of males and 100% of the females fulfilled the ISCD criteria for osteoporosis. On the other hand, all of ‘I’-RPs and ‘H’-RPs presented normal (‘I’) or slightly increased (‘H’) total (TB-BMD/Head-BMD) and increased local (PF-BMD) of the targeted joint (Figure 3). Comparing the incidence of osteoporosis reported by Borissova et al. [42] and Kirilova et al. [43] to that of our patients from identical age groups, we noted a higher rate of about 50% in females with K/L-III pHOA. We attribute this to the negative impact of immobilization on osteoporosis incidence.

### 2.2. Methods

1. **Clinical Parameters** (CPs): Clinical parameters included vital signs (blood pressure, heart rate and rhythm, breathing, and body temperature); height/weight and *BMI*; pain at walking *(WP)* by WOMAC-A and the visual analogue scale 0–100 mm (VAS-100 mm) [37]; functional ability (WOMAC-C) [38]; the presence of adverse events (AEs); therapeutic responses, based on the OMERACT-OARSI set of responder criteria [44] and minimal clinically important improvement (MCII) [45]); and quality of life (SF-36 and PtGA) [46].
2. **Laboratory parameters** (LPs), including:
  ✓ *Safety tests*: blood count, blood sugar, liver functional tests (aspartate aminotransferase—AsAt), gamma glutamate trans peptidase—gGTP, total bilirubin, and kidney functional tests (BUN, serum creatinine);
  ✓ *Serum phosphate levels*: total and ionized calcium levels;
  ✓ *Serum 25-hydroxy vitamin D* (25-OH-D): chemiluminescent immunoenzymatic assay (CLIA/reference range 20–120 ng/mL/) [47];
  ✓ *Plasma EDTA levels of intact (whole) parathyroid hormone (iPTH)*: automated electrochemiluminescent immunoenzymatic assay analyzer (Modular Analytics E170; Roche Diagnostic GmbH, Mannheim, Germany, reference range 15–65 pg/mL or ng/L) [48];
  ✓ *Serum β-beta-isomerized carboxy-terminal cross-linking telopeptide of type I collagen (sCTX I*/*β-Cross Laps*): a marker of bone turnover (BT) [47,49], CLIA methodology (reference range: men > 60 years old < 0.7 ng/mL; women > 60 years old (postmenopausal) <0.9 ng/mL).
  ✓ *Urine C-terminal crosslinking telopeptides of collagen type II (uCTX-II)*: a marker of cartilage turnover (CT) [50,51] (competitive ELISA, Cartilaps, IDS, Boldon, UK, (reference range 129 and 345 ng/mmol Cr), with intra- and inter- assay CVs below 8% and 10%, respectively). The urine concentration of CTX-II (ng/L) was standardized to total urine creatinine (mmol/L), and the units for the corrected uCTX-II concentration were ng/mmol [50].
3. **Musculoskeletal ultrasound** (MSUS), Gray scale US (GSUS), was used to measure the distance between the femoral neck and the joint capsule in both the target and the contralateral joint (the presence of an effusion and synovitis was one of the exclusion criteria), assess the bone profile and the bursae adjacent to the hip joint, and detect the presence of CPPD deposits in the labral cartilage. We used the power Doppler US (PDUS) in all cases of joint effusion or synovial hypertrophy (GSUS) detection to evaluate changes. The Esaote-MyLab-6 US machine, equipped with a 1.5–8.5 MHz convex and 3–13 MHz linear probe, was used for all MSUM evaluations. The clinical and MSUM follow-up and the therapeutic interventions under US guidance were performed by rheumatologists, certified by EULAR.
4. **DXA measurements** were performed on Lunar Prodigy Primo-en CORE, version 17, trabecular bone score (TBS) upgrade, according to ISCD methodology from 2015 [52]. The following parameters were assessed:
  ✓ *Bone geometry* (hip axis length (HAL); neck shaft angle (NSA); minimal neck width (MNW));
  ✓ *Bone mineral density* (BMD)—regional (lumbar spine BMD/LS-BMD); proximal femur BMD (PF-BMD) and total BMD (total body (TB-BMD));
  ✓ *Body composition* analyses (BCA).
5. **Radiologic investigation and parameters:** All radiographs (anterior–posterior, weight bearing dual hip) were performed on SIEMENS Axiom Iconos R200-digital, 2010 (pixel spacing 100 μm.), in an erected (weight-bearing) position at a distance of 100 cm from the source, with the ray directed perpendicular to the object and focused at 4 cm. above the symphysis under a slight internal rotation (15°) of the feet, provided by a ‘V’- shaped footrest. According to recommendations of the Consensus group from Barcelona [53] and OARSI [54], the following parameters were assessed:
  ✓ *Radiologic grade (RG)*, according to the Kellgren-Lawrence scale (K/L-grade) [39];
  ✓ *Radiologic pattern (RP),* according to classifications of Solomon/Bombelli/OARSI [13,14,55];
  ✓ *Mean joint space width (mJSW)*, determined as a mean value in three separate points (superolateral, superomedial, and apical), measured with software for digital radiologic images [53,54,55];
  ✓ *Zone of maximal joint space narrowing (max -JSN)* and closely associated *FHM (femoral head migration pattern*) [53,54,55];
  ✓ *Annual rate of joint space narrowing (JSN mm/yearly)*, calculated as differences in the values of mJSW, measured every 12 months (M0 (baseline)–M12; M12 –M24; M24–M36; M36–M48; M48–M60; and M60–M72) [53,54,55].

Radiologic investigations (X-Ray/DXA) were conducted by two separate radiologists, additionally certified by the ISCD for conducting DXA/QCT investigations, with very good inter-reader reliability (intraclass correlation coefficient ICC of 0.918, 95% CI: 0.846–0.960) and PABAK (prevalence-adjusted and bias-adjusted kappa) values for X-ray/DXA readings of 0.860 and 0.880, respectively.

6. **Selection of variables, referring to the classification:** baseline identification of EF (M0) 4 groups of indicators were used:
  ✓ *Demographics*: Due to the elimination of the influence of the factors age, BMI, malalignment on the progression of the disease as a result of our design, demographic factors included in the baseline identification of the EF groups were *sex* and *disease duration*.
  ✓ *Clinical indicators*: Pain during walking (WOMAC-A) and functional mobility (WOMAC-C) were integrated into the composite WOMAC score as the sum of WOMAC-A and WOMAC-C.
  ✓ *Radiologic indicators*: These included *RG* (K/L), *RP* (Bombelli/OARSI), *mJSW,* and *max-JSN*. During the classification process at M36, mJSW was replaced by JSN mm/yrs. (annual rate/speed of joint space narrowing) as an indicator, determined as the difference between the values of mJSW, measured every 12 months (mJSW-M0–mmJSW-M12; mJSW-M12–mmJSW-M24 etc.). The changes in JSN are discordant (opposite) to the mJSW values, but according to published sources, this indicator is more sensitive to time changes. [53,54,55].
  ✓ *Laboratory indicators*: The classification process included the following laboratory factors, taking into account the specificities of the studied population and the research design: *sCTX-I* and *uCTX-II* (concordant changes in their levels are shown in Figure 3). These were integrated into the composite biomarker score, which was calculated as sCTX-I pg/mL + uCTX-II ng/mmol Cr. The *25-OH-D* (M0) levels were included in the baseline descriptive analyses, but not in the composite variable biomarker score because vitamin D supplementation in all the groups (part of the SC) reached the target levels of ≥60 ng/mL prior to the onset of the studied therapies (M01) (Figure 2).
  ✓ *DXA parameters*: The variables TB-BMD, LS-BMD, and PF-BMD were integrated into a composite BMD score g/cm^3^ (TB-BMD+LS-BMD+PF-MD) because of their concordance in separate radiographic patterns.

In the classification process, cut-off values were identified for each of these sets of variables and were used to determine the phenotype membership of each subject. Where possible, validated cut-offs already used in OA research or clinical practice were adopted (e.g., malalignment of the Valgus or Varus ≥ 2°). Where existing cut-offs were not available, specific values were determined based on the variable distribution in the selected sample. For example, a concentration higher than the 25th and lower than the 75th percentile of each of the biomarkers was used as a specific cut-off to determine the membership to the ‘A’-RP or ‘H’-RP.

**Statistical methods**

We conducted a non-supervised hierarchical agglomerative clustering analysis using Ward’s linkage method and the squared Euclidean distance [56]. A clustering analysis was performed with no imputation on missing values. Cluster similarity and overlapping were controlled with a dimension reduction method based on multidimensional scaling [57]. The optimal number of clusters was determined and confirmed by a combination of statistical criteria, such as measurements of the within-cluster sum of squares and the gap statistic method [57,58,59]. We assessed the stability of the clustering partition using a nonparametric resampling method with 250 bootstrap samples, and calculated the clusterwise Jaccard index dispersion [59,60]. Finally, the significance of our clustering was assessed with the Gaussian null hypothesis test with a family-wise error rate of <0.05 [61]. We performed a descriptive analysis and comparison of the different clusters. Quantitative variables were analyzed with the non-parametric Kruskal–Wallis test. The significance threshold was set at *p* < 0.05 for statistical analysis. A Holm-Bonferroni method was applied to adjusted *p*-values for correction to ensure the robustness of our results and to limit the bias associated with the multiplicity of statistical tests [62]. Features comparing clusters were analyzed using as a reference the pauci-symptomatic cluster 1 with the Wilcoxon-Mann-Whitney test and Pearson’s chi-square test for quantitative and categorical variables, respectively. Univariate and multivariate multinomial logistic regression analyses were performed to examine the impact of gender and FHM on the clinical and radiographic progression of pHOA.

## 3. Results

**Figure 4:**
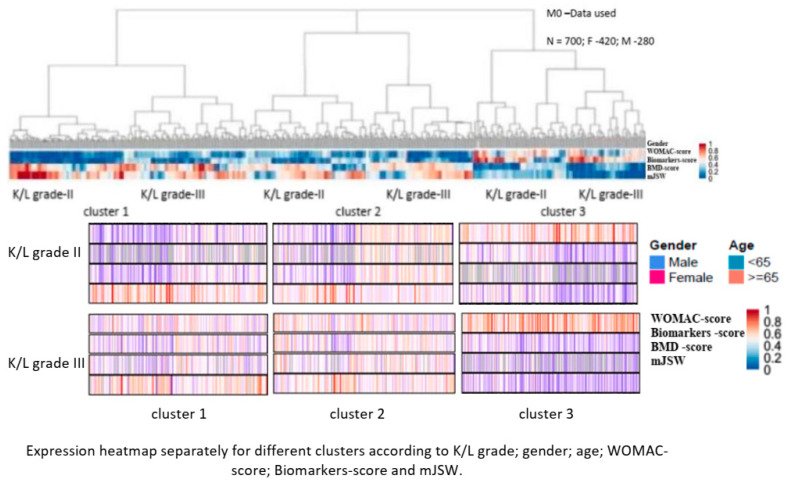
Hierarchical agglomerative clustering at baseline (M01), using input data from both studies. Dendrogram and expression heatmap of each cluster based on WOMAC score (WOMAC-A+WOMAC-C), biomarkers score (s-CTX-I + u-CTX -II), and mean joint space width (mJSW). For each individual, high scores are in red and low scores are in blue.

**Figure 5:**
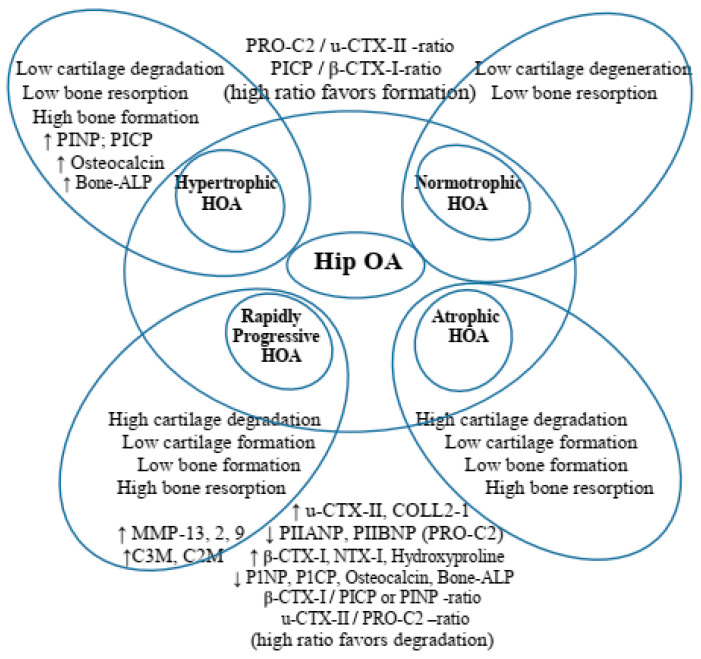
pHOA endophenotypes at baseline, using input data from both studies. PICP/PINP: ‘C’ and ‘N’ propeptides of type I collagen; bone ALP, a bone-specific alkaline phosphatase; u-CTX-II, a urine C-terminal crosslinking telopeptide of collagen type II; s-CTX-I serum beta-isomerized carboxy-terminal cross-linking telopeptide of type I collagen; PIIANP/PIIBNP, a N-terminal pro-peptide of types IIA (embryonic variant) and IIB (variant present in mature articular cartilage); PRO-C2 assay, which measures only released PIIBNP (N-terminal propeptide of mature articular cartilage), which is cleaved at maturation, and its measurement as PIIANP also reflects cartilage formation; COLL 2-1 assay, targeting an epitope located in the N-terminal triple helical region of the 3/4 fragment, a marker of degradation of type II collagen; NTX-I, an N-terminal telopeptide of collagen type I marker of bone resorption; MMP (matrix metalloproteinases) 13, 2, 9, and 3; C1M/C2M/C3M—MMP-degraded type I, II, and III collagen; C1M, which detects MMP 2, 9, and 13-generated type I collagen fragment; C2M, which detects an MMP-9-derived fragment of type II collagen fragment; C3M detecting MMP-9-derived type III collagen fragment; and ‘RP-HOA’, not present in our studies but retained in the figure in order to visualize differences with ‘A-HOA’.

The primary EF (M01) was identified using four groups of indicators: WOMAC-scores (WOMAC-A+WOMAC-C); biomarkers-scores (s-CTX-I + u-CTX-II); BMD-scores; and mJSW (Table 2 and Table 3). The identified three clusters (EFs) were closely associated with the three X-ray patterns (‘H’-RP; ‘I’-RP; and ‘A’-RP), at each of the two X-ray stages (Figure 4 and Figure 5). Patient characteristics of M01 (baseline) are shown in Table 3.

Cluster 1 (‘H’-RPs; n = 250) was characterized by the lowest levels of pain during movement and the least limitation of physical function, with the highest values for the duration of complaints (disease duration (DD)), normal 25-OH-D levels, lower-bound (below the 25th percentile) markers for bone and cartilage turnover, and increased BMD at all measurement points (LS-BMD; PF-BMD; and TB-BMD) (Table 4; Figure 4 and Figure 5).

*Cluster 2* (‘I’-RPs; n = 250) was characterized by intermediate values of pain, functional limitation, and DD. Vitamin D deficiency was found in 50% of patients with normal iPTH and forge limited (over 75th percentile) values of bone and cartilage turnover markers. Total (TB-BMD) and spine (LS-BMD) BMDs were normal, with the target joint having a normal or slightly increased regional (PF-BMD) BMD (Table 4; Figure 4 and Figure 5).

*Cluster 3* (‘A’-RPs; n = 200) was characterized by the highest values of pain during movement and functional limitation, and the lowest duration of complaints (rapid progressors). All patients had vitamin D deficiency (10 ng/mL < D levels < 20 ng/mL), and 90% of them were severely deficient (D levels < 10 ng/mL), 15% of whom had reversible, compensatory increased iPTH. There was an increase in markers of bone and cartilage turnover and a decrease in BMD at all measurement points, with 80% of patients and 100% of women meeting ISCD criteria for osteoporosis (Table 4; Figure 4 and Figure 5).

Descriptive analyses and comparisons between the main variables from the identified clusters were performed and each cluster was homogeneous and different to the others regarding the presentation of clinical symptoms (WOMAC-scores), the values of bone and cartilage turnover markers, total and regional BMD (TB-BMD; LS-BMD; and PF-BMD), as well as mean joint space width (mJSW) values (*p* < 0.05; Kruskal–Wallis test with Holm-Bonferroni post hoc analysis and correction on adjusted *p*-values)—Table 4 and Supplementary Table S1.

In addition, post hoc comparisons showed that the characteristics of the samples based on the 3 X-ray patterns (“H”; “I”; and “A”) overlapped with the characteristics of the samples of the three clusters identified 8 years later (*p* < 0.05; Kruskal–Wallis test with Holm-Bonferroni post hoc analysis and correction on adjusted *p*-values).

We repeated the classification process for the data at M36, when the specific effects of therapies on the control parameters were dropped and only the changes in the BMD score in the ZA group were retained. Temporal changes in indicators; the associations between clinical, laboratory, and radiographic changes; and the effects of individual therapies on clinical and radiographic progression have been discussed previously [31,34]:

✓ IA-CS was found to cause primary changes in WOMAC scores that were registered in M6 and were lost completely in M24. This was followed by no significant changes in s-CTX-I/u-CTX-II that were registered in M12 and lost completely in M24. No changes in JSN were registered at any time point (Table 5) [31].
✓ The main changes that IA-HA was linked to were in the WOMAC (registered in M6 and lost completely in M30), followed by no significant changes in the levels of u-CTX-II (registered in M12, lost completely in M24) and JSN (registered in M12, lost completely in M36) (Table 5) [31].
✓ ZA was associated with primary significant changes in the levels of s-CTX-I (registered in M6, lost completely in M36), followed by no significant changes in WOMAC scores (M12; M24, lost completely in M36), significant changes in BMD scores (M12; M24; M36), and significant JSN changes for ‘A’-RPs and for 60% of ‘I’-RPs (registered in M12, lost completely in M36) (Table 5) [34].

**Figure 6:**
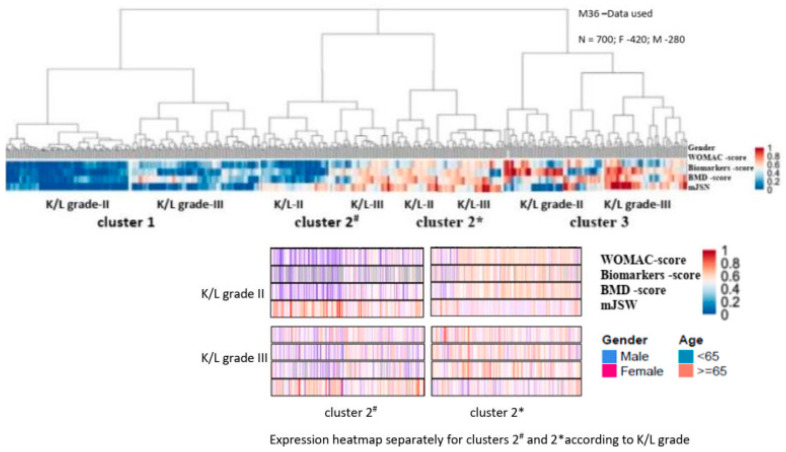
Repeated hierarchical agglomerative clustering using the M36 data from both studies. Dendrogram and expression heatmap of each cluster based on WOMAC score (WOMAC-A+WOMAC-C), biomarkers score (s-CTX-I + u-CTX -II), and mean joint space narrowing (mJSN). For each individual, high scores are in red and low scores are in blue.

**Figure 7:**
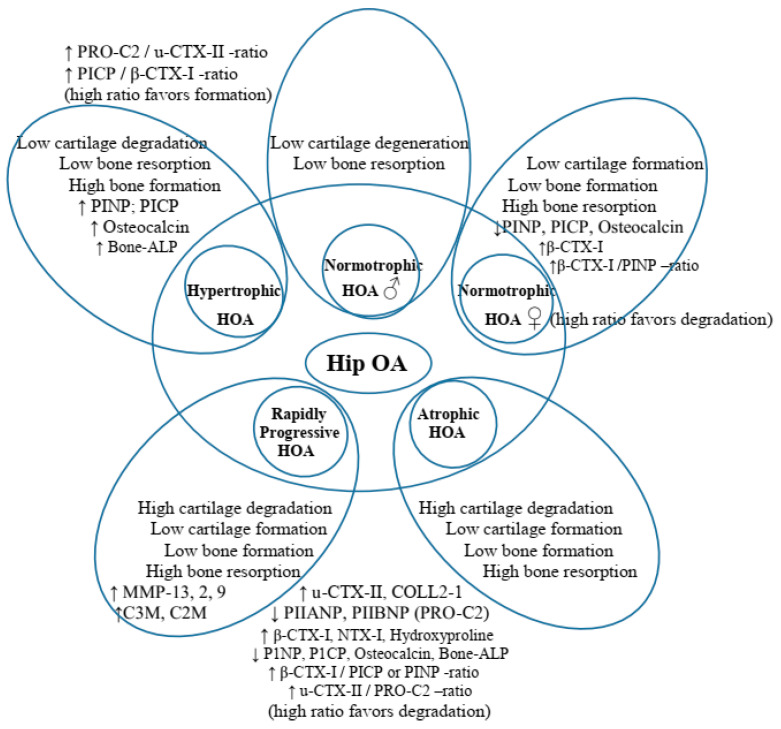
Hierarchical agglomerative clustering at M36 using data from both studies. PICP/PINP: ‘C’ and ‘N’ propeptides of type I collagen; Bone ALP: bone-specific alkaline phosphatase; u-CTX-II: urine C-terminal crosslinking telopeptides of collagen type II; s-CTX-I: serum beta-isomerized carboxy-terminal cross-linking telopeptide of type I collagen; PIIANP/PIIBNP: N-terminal pro-peptide of types IIA (embryonic variant) and IIB (variant present in mature articular cartilage); PRO-C2: assay that measures only released PIIBNP (N-terminal propeptide of mature articular cartilage), which is cleaved at maturation, and its measurement also as PIIANP reflects cartilage formation; COLL 2-1: assay, targeting an epitope located in the N-terminal triple helical region of the 3/4 fragment, a marker of degradation of type II collagen; NTX-I: N-terminal telopeptide of collagen type I marker of bone resorption; MMP (matrix metalloproteinases) 13, 2, 9, and 3; C1M/C2M/C3M: MMP degraded type I, II, and III collagen; C1M detects MMP-2, 9, and 13-generated type I collagen fragment; C2M detects an MMP-9-derived fragment of type II collagen fragment; C3M detects MMP-9-derived type III collagen fragment; and ‘RP-HOA’, not present in our studies, but retained in the figure in order to visualize differences with ‘A-HOA’.

As anticipated, the secondary classification process retained Cluster 1 and Cluster 3, with a complete overlap of ‘H’-PRs and ‘A’-RPs from both studies (similar to M0). However, the sex distribution in the primary ‘I’-RPs pool of the two studies unexpectedly led to the splitting of Cluster 2 into two subgroups: cluster 2* associated with female patients and cluster 2# associated with male patients (Figure 6 and Figure 7).

*Cluster 1* (‘H’-RPs; N = 250) retained the characteristics of the baseline (M0) with the lowest values of pain during movement and the weakest limitation of physical function, with the highest values for disease duration (DD), lower-bound (below the 25th percentile) markers for bone and cartilage turnover, and increased BMD in all measurement points (LS-BMD; PF-BMD; and TB-BMD) (Table 5 and Table 6; Figure 6 and Figure 7). Slow progressors included SC, IA-CS, and ZA from K-LIII finished with planned THR-M60; IA-HA-M66, K-LII-SC, IA-CS, and ZA finished at M84; and IA-HA at M96.

*Cluster 3* (‘A’-RPs; n = 200) also retained baseline characteristics (M0), with the highest values of pain during movement and functional limitation, and the lowest DD. Despite the early (M01) compensated D-deficiency, markers of bone and cartilage turnover were again the highest at M36, and BMD was reduced again at all measurement points (Table 6: Figure 6 and Figure 7). Fast progressors: K-LIII patients who dropped out in M 37 (SC); M42 (ZA/IA-HA) and M54, respectively (CS); M60 (IA-HA); M66 (ZA) (Table 6 and Table 7; Figure 6 and Figure 7).

*Cluster 2* (‘I’-RPs; n = 250), which had intermediate values of pain, functional limitation, DD, and D deficiency in 50% of patients with normal iPTH and forge limited (over 75th percentile) values of markers for bone and cartilage turnover, was evolutionarily divided into two subgroups:

Cluster 2* was associated with female patients (n = 150) with rapid progressors, including K-LIII undergoing planned THR at M42 (SC/IA-CS), M54 (IA-HA; ZA), respectively; K/LII at M72 (SC; IA-CS); and M78 (ZA; IA-HA). It was characterized by normal regional (PF-BMD) BMD and osteopenia of the spine (LS-BMD) and general (TB-BMD) BMD. Despite early compensation for vitamin D-deficiency (M01), markers of bone and cartilage turnover remained forge limited (above the 75th percentile) (Table 5 and Table 6; Figure 6 and Figure 7).

*Cluster 2#* was male-associated with slow progressors, including K/LIII, which ended up with planned THR at M54 (CS; ZA; IA-CS) and M60 (IA-HA), respectively; K/LII, which graduated to M72 (SC; ZA; IA-CS); and M80 (IA-HA). It was characterized by normal total (TB-BMD) and spine (LS-BMD) BMD, and slightly increased regional (PF-BMD) BMD of the target joint (Table 5 and Table 6; Figure 6 and Figure 7).

Descriptive analyzes and comparisons were again performed (similarly to the initially identified three clusters of M0) and each cluster was homogeneous, differing from the others based on clinical symptoms (WOMAC-scores), values of bone and cartilage turnover markers, total and regional BMD, as well as mean joint space width values -*p* < 0.05 (Kruskal–Wallis test with Holm-Bonferroni post hoc analysis and correction on adjusted *p*-values)–Supplementary Tables S1 and S2.

In addition, the influence of the patients’ sex and the type of migration of the femoral head in the acetabulum (FHM) on clinical and X-ray progression of the disease was analyzed (Table 7, Table 8 and Table 9).

FHM, depending on the zone of maximum narrowing (max-JSN), was classified as superior, axial, and medial (concentric) [16,53]. In men, the superior type was more common, whereas in women and patients with bilateral disease, axial and medial FHM was more frequent [16]. In our study, the three types of FHM occurred at a similar frequency in individual EFs (Table 3 and Table 4).

After the inclusion of the variable sex (females), in addition to the superior FHM into the regression equation, the model improved with an increase in adjusted R-squared and a decrease in standard deviation.

In our group of patients, the FHM type influenced clinical and radiographic progression, which increased over time (M12 → M36), regardless of the cluster affiliation (*p* < 0.05 [95% CI]). The superior type of migration was associated with a faster progression, and female sex was an additional risk factor, further defined by cluster affiliation (Table 7, Table 8 and Table 9).

The influence of age, BMI, and malalignment on clinical and radiographic progression in individual clusters was eliminated from the design through the design exclusion criteria.

Biomarker levels of bone and cartilage turnover, and BMD were directly associated with individual clusters (*p* < 0.05) and their changes from M0 through M36 were significant only as absolute values (cluster 1M01 vs. cluster 1M36), but not as a proportion (cluster 1M36 vs. cluster 2#M36 vs. cluster 2*M36 vs. cluster 3M36).

**Figure 8:**
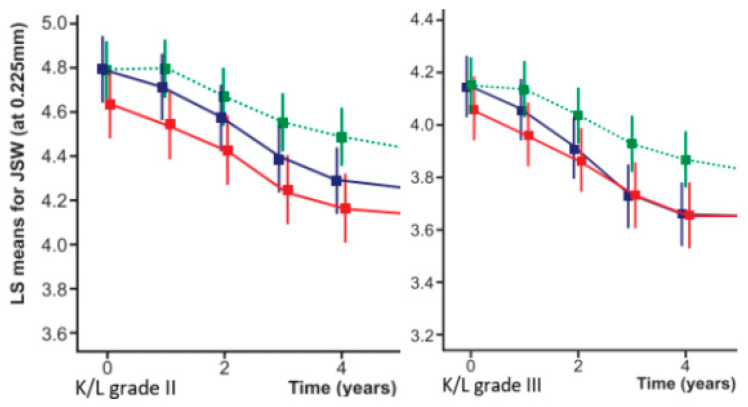
Treatment effect of IA-CS on radiological progression in diffwrent clusters: cluster 1 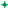; cluster 2# 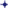; cluster 2* 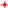; squares and error bars represent the estimated mean and the 95% confidence limits.

**Figure 9:**
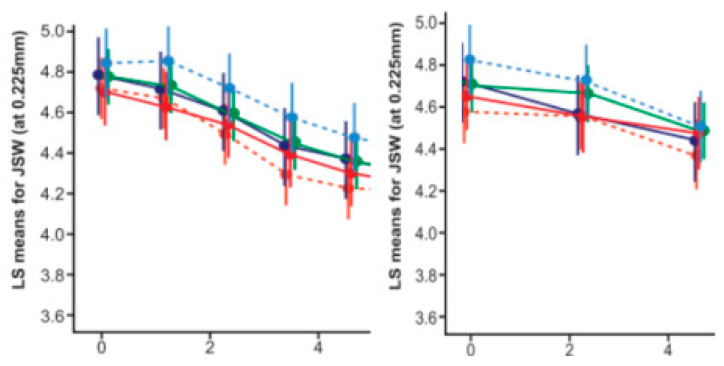
Treatment effect of IA-HA (left image) and ZA (right image) on radiographic progression in different clusters: dots/squares and error bars represent the estimated mean and the 95% confidence limits; cluster 1 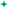; cluster 2# 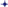; cluster 2* 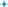; cluster 3 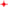; radiographic progression in SC group–cluster 3 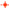.

**Figure 10:**
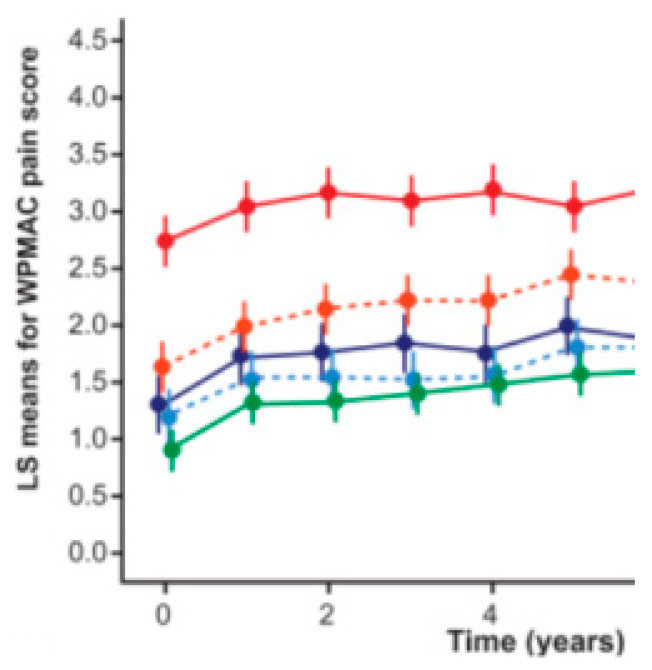
Effect of simple analgesics (paracetamol up to2.0 g/24 h), included as “rescue medication” before elective THR in cluster 1 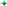; cluster 2# 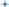; cluster 2* 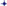; cluster 3 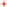; and SC group –cluster 3 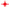, without analgesics. Dots/squares and error bars represent the estimated mean and the 95% confidence limits.

The effects of individual therapies in different ‘RPs’ on clinical and radiographic progression over time are discussed in detail in related studies [31,34]. Here we will summarize the effect of different therapies on the different clusters in the time interval from M01 (start of therapeutic interventions) through M36 (disappearance of ≥75% of the effect):

1. The effect size (ES) of ia-CS was most pronounced in cluster 1: ES-SMDCS (standardized mean differences) = 0.6 (95% CI: 0.1 to −1.1; *p* = 0.021), equal to the effect of IA-HA here, but with a faster onset (M1) and a duration of 12 to 24 months (K/L-III/K/L-II), without delayed negative effects on the BMD score/time to THR, compared to the SC group in the same cluster.
  Unidirectional and positive changes in the biomarkers ↓s-CTX-I and ↓u-CTX-II were found in this group (cluster 1) at M6 and were absent at M18, reflecting a loss of therapeutic effect at M12 (K/LIII) and M24 (K/LII). Changes in biomarkers were related, however, not to the effect of ia-CS on synovitis/hydrops (exclusion criteria in the design), but to an effect on pain, increased functional capacity, and improved trophic characteristics of the cartilage and subchondral bone, which also explained the best effect of simple analgesics and exercise therapy in this group of patients.
  In cluster 2#, changes in M12 biomarkers were multidirectional (↑s-CTX-I; ↓u-CTX-II) and were accompanied by a decrease in M36 BMD score (compared to SC) and an earlier loss of effect (M12-K/LIII; M18-K/LII). There were no negative changes in time to THR (compared to SC).
  Cluster 2* demonstrated early (M6) negative effects on the biomarkers ↑s-CTX-I and ↑u-CTX-II, accompanied by an early (M24) decrease in BMD score that persisted until the end of the follow-up, with a negative effect on the time to THR (relative to SC). No changes in mJSN were registered at any time point (Table 6 and Table 10; Figure 8) [31].
2. The effect of ia-HA was moderate (ES-SMDHA = 0.44; 95% CI: −0.1 to 1.0; *p* = 0.042), with a delayed onset (M2) in all clusters, although the rapid evolution of cluster 3 prevented its deployment.
  Unidirectional, positive (↓s-CTX-I; ↓u-CTX-II) biomarker changes were found at M6 in all clusters that were lost at M30 (cluster 1); M24 (cluster 2# and cluster 2*); and M18 (cluster 3) changes in BMD-scores (vs. SC) were absent at all-time points.
  The duration of the effect was a function of X-ray stages, and for K/L-III, it was exhausted for cluster 1 at M24 (OARSI responses) and M30 (MCII); for cluster 2# and cluster 2* at M18 (OARSI responses) and M24 (MCII); for cluster 3 at M12 (OARSI responses) and M18 (MCII). In K/LII, cluster 1 retained OARSI responses until M30 and MCII until M36; cluster 2# lost OARSI responses at M24 and MCII at M30; cluster 2* corresponded to M18 (OARSI) and M24 (MCII); cluster 3 corresponded to M12 and M18.
  Significant changes were registered in mJSN (M12), persisting until M36 (cluster 1; 2#; 2*) and M24-cluster 3 (*p* < 0.05) (Figure 9), reflecting significant differences in ‘time to THR’ (*p* < 0.001) (Table 6 and Table 10; Figure 9) [31].
3. The effect of ZA (in contrast to IA-CS) was most pronounced in cluster 2* and cluster 3, ES-SMDZA = 0.5 (95% CI: 0.1 to −1.1; *p* = 0.024), absent in cluster 1, and null in cluster 2#. There was a delayed onset corresponding to changes in biomarkers, which on M6 were unidirectional (↓s-CTX-I; → u-CTX-II), on M12 were unidirectional positive (↓s-CTX-I; ↓u-CTX-II), persisting until M24 (for all clusters) and were lost at M36 (for all clusters). In contrast, changes in BMD score (registered at M12) were most pronounced at M36 (*p* < 0.001, vs. SC), accompanied by changes in WOMAC-score (M12- MCII; M24 –OARSI responses; M36 –MCII; lost completely at M42) and significant changes (*p* < 0.001, vs. SC/IA-CS) in mJSN (registered on M12, lost completely on M48) and ‘time to THR’ (*p* < 0.001, vs. SC/IA-CS) (Table 6 and Table 10; Figure 9) [34].
4. Patients from different clusters had a different therapeutic response, both to the paracetamol analgesia used during follow-up (Figure 10), to NSAIDs (diclofenac sodium up to 150 mg/24 h), and to opioid analgesics (tramadol hydrochloride up to 200 mg/24 h). NSAIDs (diclofenac sodium 2 × 75 mg) and tramadol hydrochloride 2 × 100 mg, were allowed as a “rescue medication”, from the time of follow-up withdrawal until planned THRs were performed (on average between 20 and 40 days). The time to withdraw from follow-up and refer to elective THR was a shared decision between the principal investigator and the patient, when the level of pain and the degree of functional limitation had become unacceptable for the patient while on analgesia with paracetamol up to 2.0 g/24 h.
  Clusters 1 and 2# did not show a significant difference (*p* ≥ 0.05) in their response to different analgesics (Diclofenac 2 × 75 mg/24 h vs. Tramadol 2 × 100 mg/24 h), in contrast to cluster 2* (*p* = 0.024; in favor of tramadol hydrochloride) and cluster 3 (*p* < 0.001; in favor of tramadol hydrochloride)—descriptive analyzes and comparisons of analgesia with Diclofenac 2 × 75 mg/24 h vs. Tramadol 2 × 100 mg/24 h are presented in Table S3 of the Supplementary Material.

## 4. Discussion

A multiple factor analysis with clustering uses multiple clinical input variables to determine whether different groups of patients can be distinguished. A multifactor analysis is an extension of a principal component analysis that assigns weights to variables by balancing the influence of groups of similar variables in the global analysis.

Our study is the first population-based study on pHOA in Bulgaria with a sample size of 2200 (M0)/700 (after randomization–M01/M36), comparable to that of our population-based studies on population vitamin D status [41] and the incidence of osteoporosis and osteopenia in the country [42,43]. This study’s results have provided information about the frequency of various EF pHOAs in Bulgaria; clinical, radiographic, and laboratory characteristics of remote EFs; types of responses to therapies used so far; and rate of progression (time to THR) in individual EFs.

For the first time in our study, an overlap of cluster characteristics with those of the three X-ray models (‘H’-RPs; ‘A’-RPs; and ‘I’-RPs) used in the original design 10 years earlier has been established (*p* < 0.05; Kruskal–Wallis test with Holm-Bonferroni post hoc analysis and correction on adjusted *p*-values). This overlap is essential because, in the process of screening and recruiting the cohort, rheumatologists from the three participating centers were guided only by a target number of patients (150 patients of each model for each of the two K/L grades), based solely on the inclusion and exclusion criteria, standing (weight-bearing) position hip radiographs, and safety blood tests (blood count; liver and kidney function tests). Investigations on the levels of biomarkers (25-OH-D, s-CTX-I, u-CTX-II) and BMD assessments (DXA) were performed after collecting the cohort and dividing patients into groups according to radiographic stage and pattern (M0), shortly before the randomization process (M01).

In practice, this is the possibility of assessing the course of the disease, the balance of the processes of bone and cartilage turnover, the prediction of therapeutic responses, and even the prognosis (time to THR) based solely on history, clinical examination, and conventional X-ray imaging. Guidelines can be set for necessary investigations and follow-up periods for each cluster, which are especially important for our aim of updating the consensus on the prevention, non-surgical treatment, and surgical treatment of pHOA in our country. No less important is the fact that, with the above-mentioned physical methods and properly interpreted X-ray images, we have the perfect screening and distribution of patients (EF pools) necessary for the design of modern pHOA clinical trials.

The secondary classification process with M36 data revealed two new EFs, arising from the original cluster 2 and strongly associated with the variable sex, each with its own rate of progression and therapeutic responses. New EFs were found when following the evolution of the disease, which helped address the following questions:

1. Which patients from cluster 2 will respond well to antiresorptive therapy? In the discussion of the intravenous bisphosphonate trial [34], we noted that only a proportion (60%) of ‘I’-RPs realized a response, associating responses with patients having baseline elevated levels of markers of bone turnover and osteopenia for total and spine BMD from baseline DXA examinations. The cluster analysis with data from M36 strongly linked these data to the patients’ sex, indicating that it was the female patients of cluster 2 in whom the evolution of pHOA was associated with a progression of BMD losses over time and their negative effect on subchondral bone remodeling.

2. What impact does the FHM type have on pHOA progression? Cluster affiliation, a risk factor that affects the progression of all EFs, was not a determining factor; however, the effect was potentiated by the patients’ sex. Specifically, it was found that women from cluster 2* and cluster 3 with superior FHM should undergo frequent follow-up and therapy (bone preparation for elective THR) with intravenous bisphosphonate as early as possible.

3. Where is the place of existing therapies in individual clusters, related to the presence of quantitative and qualitative differences in the effect of therapies in individual EFs?

Cluster 1: Responds best to exercise therapy, simple analgesics, and IA-CS. For this reason, in the complex approach, measures to optimize the load and biomechanics (BMI corrections; insoles; orthoses) are taken first, followed by specialized physical therapy to balance and maintain muscle tone, and pain relief (maintenance of motor mode and quality of life) with simple analgesics and, if necessary, IA-CS. The above measures provided a quick and long-lasting (equal to the duration of IA-HA in this group) effect at a low cost, without long-term negative impact on bone and cartilage turnover and BMD.

Cluster 2: For both men and women, treatment with IA-HA (the best answer due to long enough natural evolution and time to unfold its effect) had a positive effect on clinical and X-ray progression. Men (cluster 2#) received additional benefits from physical therapy, while women (cluster 2*) experienced improvement from physical procedures with trophic action and ZA. Unfortunately, we lack a study on the effect of anti-receptor activator of the nuclear factor-κB ligand antibody (Denosumab) therapy in OA/pHOA, and this cannot be recommended at this time, despite its proven effects on radiographic progression in erosions, as well as new bone formation in inflammatory joint diseases (RA/SpA/PsA), modifying the processes in the subchondral bone [63,64,65]. IA-CS analgesia was not effective in these patients, showing delayed negative effects in C2# and early negative effects in C2* on X-ray progression.

Cluster 3: Due to a rapid natural evolution in patients in this cluster, the effect of IA-HA does not have the time to unfold; therefore, it is not recommended. The main focus should be on the early preparation of bone with ZA and the proper use of time until THR (restoring or maintaining the tone of key muscle groups), as well as physical procedures with an analgesic or trophic effect. An important feature of the group is problematic analgesia due to a poor response to simple analgesics and NSAIDs (similar to that of RP-HOA [25]). “Appropriate” analgetics (tramadol hydrochloride and oxycontin) or combination forms, in addition to the well-known adverse effects on age-specific constipation, raise the risk of falls and fractures (especially high in this group), prompting early bone preparation (ZA) and timely definitive treatment (elective THR) in the first place. Our observations and recommendations align with the official views on the planning of definitive operative treatment [66].

While IA-CS is part of the most current 2019 OARSI [67] and ACR [68] guidelines, there is disagreement regarding the extent of the effect (ES) and benefit of IA-HA and bisphosphonates (ZA) in pHOA therapy. Furthermore, OARSI and ACR’s 2019 recommendations on the subject are unfavorable. We do not dispute this point of view because it reflects current knowledge on the matter, and more recent recommendations based on DBRCT results tailored to the therapeutic responses of individual EFs are lacking. The negative opinions from 2019 [67,68] were based on post hoc analyses of studies [69,70,71] not meeting the 2015 OARSI recommendations for planning and conducting HOA studies [40], simply because the recommendations did not exist then, and later studies responding to them [72] were not analyzed. Unfortunately, endophenotyping only became the object of clinical trial designs after 2019, hence corresponding results and recommendations have not yet been released.

In summary, our study, apart from identifying the four EF pHOAs in the Bulgarian population of men and women over 60, defined the exact place of IA-CS (cluster 1), IA-HA (cluster 2# and 2*), and ZA (cluster 2* and cluster 3) in pHOA therapy.

The limitations of this present study stem from its specific goal to identify the EFs of pHOA in the Bulgarian population and to provide an update on the national consensus on the prevention, non-surgical treatment, and surgical treatment of the disease. Hence, the results and conclusions refer specifically to the Bulgarian population of men and women over 60 years of age, with all the characteristic features of this particular population. 

## 5. Conclusions

Clinical endophenotyping creates homogeneous patient groups needed for modern genetic and therapeutic studies on pHOA, while also giving doctors guidelines for how to treat each patient individually. The methodology of clinical endophenotyping should be established as part of a systematic approach for these patients, facilitating the coverage, prevention, follow-up, and treatment planning of large and homogeneous groups of patients from the same EF.

## Figures and Tables

**Table 1:** Incidence of different radiographic patterns in our sample and in Ledingham et al. [15].

Age, Sample Size, Gender Distribution	Distribution of Patterns (%)			
	This Study		Ledingham et al. (1992) [15]	
Age–Median (IQR)	63 (60–69)		70 (50–90)	
Total (n)	2200	‘I’-RPs—60% ‘H’-RPs—19% ‘A’-RPs—21%	300	‘I’-RPs—60.6% ‘H’-RPs—20.3% ‘A’-RPs—19%
Female (n)	1400	‘I’-RPs—58% ‘H’-RPs—18% ‘A’-RPs—24%	185	‘I’-RPs—60% ‘H’-RPs—18% ‘A’-RPs—22%
Male (n)	800	‘I’-RPs—62% ‘H’-RPs—20% ‘A’-RPs—18%	115	‘I’-RPs—63% ‘H’-RPs—20% ‘A’-RPs—17%

**Table 2:** Vitamin D status in our sample of 700 patients of both sexes aged 60–69 years in relation to radiographic stage and pattern of primary hip osteoarthritis.

Groups and Number	Levels of 25-OH-D (20–120 ng/mL)		
	**25-OH-D ≤ 10 ng/mL** **(Severe Deficiency)**	**10 ng/mL < 25-OH-D < 20 ng/mL** **(Deficiency)**	**25-OH-D > 20 ng/mL** **(Normal Levels)**
** Men–N = 280 **	60 (21.4%)	150 (53.6%)	70 (25%)
II-‘A’–N = 40	26 (8.3%)	14 (5%)	0
II-‘I’–N = 50	0	50 (17.9%)	0
II-‘H’–N = 50	0	11 (3.9%)	39 (14%)
III-‘A’–N = 40	34 (13.1%)	6 (2.1%)	0
III-‘I’–N = 50	0	50 (17.9%)	0
III-‘H’–N = 50	0	19 (6.8%)	31 (11%)
** Women–N = 420 **	124 (29.5%)	236 (56.2%)	60 (14.3%)
II-‘A’–N = 60	60 (14.3%)	0	0
II-‘I’–N = 75	0	75 (17.8%)	0
II-‘H’–N = 75	0	41 (9.8%)	34 (8.1%)
III-‘A’–N = 60	60 (14.3%)	0	0
III-‘I’–N = 75	4 (0.9%)	71 (16.9)	0
III-‘H’–N = 75	0	49 (11.7)	26 (6.2%)

**Table 3:** Main characteristics of the sample before the classification process.

Variable	N	Median	IQR
Age	700	64	60.5–69
Disease duration (yrs.)	700	3.5	1.0–7.0
Sex (female)	420		
Sex (male)	280		
BMI (kg/m^2^)	700	25.0	21.5–28
RG (K/L) and RP			
K/L-II	350		
‘H’-RP	125		
‘I’-RP	125		
‘A’-RP	100		
K/L-III	350		
‘H’-RP	125		
‘I’-RP	125		
‘A’-RP	100		
Site of max-JSN (pattern of FHM)	700		
Superior	462 (66%); F-60%; M-75%		
Axial	91 (13%); F-15%; M-10%		
Medial	147 (21%); F-25%; M-15%		
WOMAC-A	700	8	6.0–10.0
WOMAC-C	700	37	30–45
25-OH-D (ng/mL)	700	27	6.5–65.5
s-CTX-I (ng/mL)	700	0.86	0.5–1.4
u-CTX-II (ng/mmol Cr)	700	403	240–600
mJSW (mm.)	700	3.9	3.0–4.8
Total body BMD (g/cm^2^)	700	1.37	1.0–1.8
Total hip BMD (g/cm^2^)	700	0.75	0.4–1.2
APS L1–L4 BMD (g/cm^2^)	700	0.95	0.5–1.5
Treatment groups	700		
Standard of care (SC)	300		
ZA + SC	150		
ia-HA + SC	150		
ia-CS + SC	100		

IQR: interquartile range; BMI: body mass index; RG (K/L): radiological grade according to Kellgren-Lawrence grading scale; RP: radiological pattern according to Bombelli/OARSI-atlas; WOMAC-A –WOMAC: pain scale (0–20); WOMAC-C –WOMAC: function scale (0–68); 25-OH-D: level of 25-hydroxy vitamin D; s-CTX-I: serum-beta-isomerized carboxy-terminal cross-linking telopeptide of type I collagen; u-CTX-II: urine-C-terminal crosslinking telopeptides of collagen type II, given as the corrected concentration of uCTX-II for urinary creatinine concentration in ng/mmol Cr.; mJSW: mean joint space width; APS/L1–L4/BMD: anterior–posterior spine (L1–L4) bone mineral density; ZA: zoledronic acid treatment group; ia-HA: intra-articular hyaluronic acid treatment group; ia-CS: intra-articular corticosteroid treatment group; and FHM: patterns of femoral head migration within the acetabulum in relation to the site of maximal joint space narrowing.

**Table 4:** Characteristics of the samples distributed after the classification process in the identified three clusters (EFs), completely overlapping the characteristics of the samples of the three X-ray patterns (‘H’; ‘I’; and ‘A’).

Variable	Cluster 1 (‘H’-RPs) Values—Median (IQR)	Cluster 2 (‘I’-RPs) Values—Median (IQR)	Cluster 3 (‘A’-RPs) Values—Median (IQR)
Pattern of femoral head migration			
Superior	67%	65%	63%
Axial	12%	13%	14%
Medial	21%	21%	23%
Age (yrs.) K/L-II	64 (62–66)	63 (61–65)	61 (60–63)
K/L-III	67 (64–69)	66 (63–68)	64 (63–66)
DD (yrs.) K/L-II	4 (3–5)	3 (2–4)	1.5 (1–2)
K/L-III	6 (4–8)	5 (4–6)	2 (1–3)
Gender K/L-II	F-75; M-50	F-75; M-50	F-60; M-40
K/L-II	F-75; M-50	F-75; M-50	F-60; M-40
BMI (kg/m^2^) K/L-II	25.5 (23.5–27.5)	25 (23–27)	24 (22–26)
K/L-III	26.5 (25.5–28)	26 (24–28)	23 (22–24)
WOMAC-A			
K/L-II	6.5 (6.0–7.0)	7.0 (6.5–7.5)	8.0 (7.5–9.0)
K/L-III	7.5 (7.0–8.0)	8.0 (7.5–8.5)	9.5 (9.0–10)
WOMAC-C K/L-II	32 (30–34)	34 (33–35)	37 (35–39)
K/L-III	38 (36–40)	41 (39–43)	44 (43–45)
WOMAC-score (A+C) K/LII	38.5 (36–41)	41 (39.5–42.5)	45 (42.5–48)
WOMAC-score (A+C) K/LIII	45.5 (43–48)	48 (46.5–51.3)	53.5 (52–55)
25-OH-D (ng/mL) K/L-II	40 (16.5–65.5)	29 (9–59)	17 (7.5–25)
K/L-III	38 (15–61)	25 (8–52)	11 (6.5–16.5)
s-CTX-I (ng/mL) K/L-II	0.6 (0.5–0.8)	0.8 (0.6–1.0)	1.0 (0.9–1.1)
K/L-III	0.7 (0.6–0.9)	0.9 (0.7–1.1)	1.2 (1.0–1.4)
u-CTX-II (ng/mmol Cr.) K/L-II	280 (240–320)	340 (320–360)	400 (360–440)
K/L-III	380 (350–420)	460 (430–490)	560 (520–600)
Biomarkers-score K/LII	281 (241–321)	341 (321–361)	401 (361–441)
Biomarkers-score K/LIII	381 (351–421)	461 (431–491)	561 (521–602)
mJSW (mm.) K/L-II	4.6 (4.4–4.8)	4.4 (4.2–4.6)	4.2 (4.0–4.4)
K/L-III	3.6 (3.4–3.8)	3.4 (3.2–3.6)	3.2 (3.0–3.4)
TB-BMD (g/cm^2^) K/L-II	1.6 (1.4–1.8)	1.4 (1.3–1.5)	1.3 (1.2–1.4)
K/L-III	1.5 (1.3–1.6)	1.3 (1.2–1.4)	1.1 (1.0–1.2)
TH-BMD (g/cm^2^) K/L-II	1.1 (1.0–1.2)	0.8 (0.7–0.9)	0.6 (0.5–0.7)
K/L-III	1.0 (0.9–1.1)	0.7 (0.6–0.8)	0.5 (0.4–0.6)
APS L1–L4 BMD (g/cm^2^) K/L-II	1.3 (1.1–1.5)	1.1 (0.9–1.3)	0.9 (0.8–1.0)
K/L-III	1.0 (0.9–1.2)	0.8 (0.6–1.0)	0.6 (0.5–0.7)
BMD-score K/LII	4.0 (3.5–4.5)	3.3 (2.9–3.7)	2.8 (2.5–3.1)
BMD-score K/LIII	3.5 (3.1–4.0)	2.8 (2.4–3.2)	2.2 (1.9–2.5)

DD: disease duration; K/L-II/K/L-III: radiological grade II/III according to Kellgren-Lawrence grading scale; BMI: body mass index; WOMAC-A/C –WOMAC: pain/function scale; 25-OH-D: level of 25-hydroxy vitamin D; s-CTX-I: serum-beta-isomerized carboxy-terminal cross-linking telopeptide of type I collagen; u-CTX-II: urine-C-terminal crosslinking telopeptides of collagen type II; mJSW: mean joint space width; TB-BMD: total body BMD; TH-BMD: total hip BMD; and APS L1–L4 BMD: anterior–posterior spine L1–L4 BMD.

**Table 5:** Changes in tracked indicators over time (M01–M36).

Cluster 1	Mean Values IQR (K/l-II+K/L-III)	M0	M6	M12	M24	M36
SC	WOMAC-score	82 (79–84)	82 (80–84)	83 (82–84)	84 (83–85)	85 (84–86)
	Biomarkers-score	662 (592–742)	670 (630–750)	690(650–760)	730 (690–770)	800 (770–850)
	BMD–score (kg/m^2^)	7.5 (7.0–8.5)	NA	7.4 (7.0–8.0)	7.2 (6.8–7.7)	7.0 (6.5–7.5)
	mJSW/mm	8.2 (7.8–8.6)	NA	7.7 (7.6–7.8)	7.0 (6.9–7.2)	6.2 (6.0–6.4)
ia-CS	WOMAC-score	82 (79–84)	78 (76–80) * ^#^	79 (77–82) * ^#^	81(80–83) ^#^	85 (84–86)
	Biomarkers-score	662 (592–742)	675 (615–755)	690 (650–750)	735 (685–775)	795(740–840)
	BMD–score (kg/m^2^)	7.5 (7.0–8.5)	NA	7.4 (7.1–8.1)	7.2 (6.8–7.6)	7.0 (6.5–7.4)
	mJSW/mm	8.2 (7.8–8.6)	NA	7.7 (7.6–7.8)	7.0 (6.9–7.2)	6.2 (6.0–6.4)
ia-HA	WOMAC-score	82 (79–84)	79 (77–81) * ^#^	78 (77–80)* ^#^	79 (79–81) * ^#^	82 (81–83) * ^#^
	Biomarkers-score	662 (592–742)	665 (610–745)	680 (650–720)	720 (685–750)	790 (730–820)
	BMD–score (kg/m^2^)	7.5 (7.0–8.5)	NA	7.4 (7.1–8.1)	7.2 (6.9–7.7)	7.0 (6.5–7.5)
	mJSW/mm	8.2 (7.8–8.6)	NA	7.8 (7.7–7.9)	7.3 (7.2–7.4) ^#^	6.7 (6.6–6.8) ^#^
ZA	WOMAC-score	82 (79–84)	82 (80–84)	82 (82–84)	83 (83–85)	85 (84–86)
	Biomarkers-score	662 (592–742)	650 (600–700)	660 (630–700)	680 (670–720) ^#^	750 (720–790) ^#^
	BMD–score (kg/m^2^)	7.5 (7.0–8.5)	NA	7.5 (7.3–7.7)	7.4 (7.0–7.7)	7.2 (6.8–7.6)
	mJSW/mm	8.2 (7.8–8.6)	NA	7.7 (7.6–7.8)	7.0 (6.9–7.2)	6.2 (6.0–6.4)
**Cluster 2#**						
SC	WOMAC-score	83 (82–84)	83 (82–84)	84 (83–85)	85 (84–86)	86 (85–86)
	Biomarkers-score	730 (700–800)	740 (710–770)	755 (730–785)	790 (765–820)	860 (830–900)
	BMD–score (kg/m^2^)	6.6 (6.1–7.1)	NA	6.5 (6.0–7.0)	6.4 (5.9–6.9)	6.2 (5.7–6.7)
	mJSW/mm	7.8 (7.5–8.1)	NA	7.2 (7.0–7.4)	6.4 (6.2–6.6)	5.6 (5.5–5.6)
ia-CS	WOMAC-score	83 (82–84)	80 (79–81) * ^#^	80 (80–82) * ^#^	85 (85–86)	87 (86–88)
	Biomarkers-score	730 (700–800)	742 (715–775)	760 (735–790)	800 (770–830)	880 (860–910)
	BMD–score (kg/m^2^)	6.6 (6.1–7.1)	NA	6.5 (6.0–7.0)	6.3 (5.8–6.6)	6.1 (5.7–6.5)
	mJSW/mm	7.8 (7.5–8.1)	NA	7.2 (7.0–7.3)	6.3 (6.1–6.5)	5.2 (5.1–5.4)
ia-HA	WOMAC-score	83 (82–84)	80 (78–81) * ^#^	79 (78–82) * ^#^	80 (80–82) * ^#^	83 (82–84) ^#^
	Biomarkers-score	730 (700–800)	736 (710–800)	740 (700–780)	775 (740–810)	850 (800–900)
	BMD–score (kg/m^2^)	6.6 (6.1–7.1)	NA	6.5 (6.1–7.0)	6.4 (6.0–6.8)	5.2 (5.0–5.4)
	mJSW/mm	7.8 (7.5–8.1)	NA	7.4 (7.2–7.5)	6.7 (6.5–6.8) ^#^	5.7 (5.6–5.8)
ZA	WOMAC-score	83 (82–84)	83 (82–84)	83 (82–84)	84 (82–84)	86 (85–86)
	Biomarkers-score	730 (700–800)	715 (680–750)	700 (680–730) ^#^	690 (680–720) * ^#^	730 (720–750) ^#^
	BMD–score (kg/m^2^)	6.6 (6.1–7.1)	NA	6.7 (6.2–7.2)	6.8 (6.4–7.1)	6.8 (6.3–7.0)
	mJSW/mm	7.8 (7.5–8.1)	NA	7.2 (7.0–7.4)	6.5 (6.4–6.6)	5.6 (5.4–5.8)
Cluster 2*						
SC	WOMAC-score	84 (83–85)	84 (83–85)	85 (84–86)	86 (85–87)	87 (86–88)
	Biomarkers-score	835 (805–905)	850 (820–870)	870 (840–890)	910 (885–935)	980 (935–1025)
	BMD–score (kg/m^2^)	5.6 (5.1–6.1)	NA	5.5 (5.0–6.0)	5.4 (5.0–5.8)	5.2 (4.8–5.6)
	mJSW/mm	7.7 (7.4–8.0)	NA	6.9 (6.7–7.1)	5.8 (5.7–5.9)	4.5 (4.4–4.6)
ia-CS	WOMAC-score	84 (83–85)	81 (82–83) * ^#^	81 (81–83) * ^#^	86 (86–87)	87 (87–88)
	Biomarkers-score	835 (805–905)	865 (835–895)	900 (875–930)	960 (945–985)	1070 (1000–1150)
	BMD–score (kg/m^2^)	5.6 (5.1–6.1)	NA	5.4 (4.9–5.9)	5.2 (4.8–5.4)	5.0 (4.6–5.4)
	mJSW/mm	7.7 (7.4–8.0)	NA	6.8 (6.6–7.0)	5.6 (5.4–5.8)	4.1 (4.0–4.2) ^#^
ia-HA	WOMAC-score	84 (83–85)	82 (82–84)	81(80–82)* ^#^	82 (81–83) ^#^	84 (83–85) ^#^
	Biomarkers-score	835 (805–905)	850 (820–870)	870 (840–890)	900 (860–920)	900 (880–950)
	BMD–score (kg/m^2^)	5.6 (5.1–6.1)	NA	5.5 (5.0–6.0)	5.4 (5.0–5.8)	5.2 (4.9–5.5)
	mJSW/mm	7.8 (7.5–8.1)	NA	7.1 (7.0–7.2)	6.2 (6.0–6.3)	4.9 (4.8–5.0)
ZA	WOMAC-score	84 (83–85)	84 (83–85)	83 (82–81)	82 (81–83)	84 (83–85)
	Biomarkers-score	790 (760–840)	760 (730–800) ^#^	720 (700–750) * ^#^	700 (680–720) * ^#^	780 (760–800) ^#^
	BMD–score (kg/m^2^)	5.6 (5.1–6.1)	NA	5.9 (5.4–6.3) * ^#^	6.4 (6.0–6.8) * ^#^	6.5 (6.1–6.9) * ^#^
	mJSW/mm	7.7 (7.4–8.0)	NA	7.0 (6.9–7.2)	6.0 (5.8–6.2)	4.9 (4.8–4.9) ^#^
**Cluster 3**						
SC	WOMAC-score	85 (84–86)	85 (84–86)	86 (85–87)	87 (86–87)	88 (87–88)
	Biomarkers-score	960 (890–1040)	975 (925–1065)	1005 (965–1090)	1070 (1020–1120)	1200 (1100–1300)
	BMD–score (kg/m^2^)	5.0 (4.5–5.5)	NA	4.8 (4.3–5.3)	4.6 (4.1–5.0)	4.2 (3.8–4.6)
	mJSW/mm	7.4 (7.0–7.8)	NA	6.4 (6.2–6.5)	5.2 (5.0–5.2)	3.7 (3.6–3.8)
ia-CS	WOMAC-score	NA)	NA	NA	NA	NA
	Biomarkers-score	NA	NA	NA	NA	NA
	BMD–score (kg/m^2^)	NA	NA	NA	NA	NA
	mJSW/mm	NA	NA	NA	NA	NA
ia-HA	WOMAC-score	85 (84–86)	83 (81–85)	82 (81–83) * ^#^	83 (82–85) ^#^	85 (84–86) ^#^
	Biomarkers-score	962 (882–1043)	970 (900–1050)	985 (940–1060)	1020 (980–1055)	1050 (1000–1100)
	BMD–score (kg/m^2^)	5.0 (4.5–5.5)	NA	4.8 (4.4–5.3)	4.6 (4.2–5.0)	4.3 (3.9–4.7)
	mJSW	7.4 (7.0–7.8)	NA	6.5 (6.4–6.6)	5.5 (5.4–5.5) ^#^	4.2(4.0–4.4) ^#^
ZA	WOMAC-score	85 (84–86)	85 (84–86)	84 (83–85)	83 (83–81)	85 (84–86)
	Biomarkers-score	962 (882–1043)	920 (880–950) *	840 (800–900) *	750 (710–780) *	800 (750–850) * ^#^
	BMD–score (kg/m^2^)	5.0 (4.5–5.5)	NA	5.2 (4.7–5.6)	5.5 (5.1–5.9) * ^#^	5.6 (5.2–5.9) * ^#^
	mJSW/mm	7.4 (7.0–7.8)	NA	6.5 (6.3–6.7)	5.4 (5.2–5.6)	4.1 (4.0–4.3) ^#^

*: indicates significant differences (*p* < 0.05) between different time points within the same group; #: indicates significant differences (*p* < 0.05) versus the standard of care (SC) group. The Kruskal–Wallis test was applied with Holm-Bonferroni post hoc analysis and correction on adjusted *p*-values; ia-CS: intra-articular glucocorticosteroids; ia-HA: intra-articular hyaluronic acid; ZA: zoledronic acid.

**Table 6:** Descriptive analyses of the four composite variables and time to THR for the two new clusters identified in M36 from the source Cluster 2.

Baseline Cluster 2 (N = 250; F-150; M-100)	Cluster 2# Median (IQR) (N = 100; K/LII-50; K/LIII-50)	Cluster 2* Median (IQR) (N = 150; K/LII-75; K/LIII-75)	*p*-Value ^†^ Cluster 2# vs. Cluster 2*
** K/L-II–N = 125 (F75/M50) **	N = 50	N = 75	
** SC–N = 50 **	**N = 20**	**N = 30**	
WOMAC-score	76 (75–77)	79 (78–80)	0.044
Biomarkers-score	401 (350–492)	485 (420–575)	0.041
BMD-score	3.3 (3.0–3.6)	2.9 (2.6–3.1)	0.442
mJSN	0.5 (0.45–0.55)	0.7 (0.6–0.8)	0.043
Time to THR	M72	M60	0.001
** ZA–N = 25 **	**N = 10**	**N = 15**	
WOMAC-score	75 (74–76)	73 (71–74)	0.044
Biomarkers-score	355 (300–410)	395 (340–440)	0.044
BMD-score	3.5 (3.3–3.8)	3.4 (3.2–3.6)	NS
mJSN	0.45 (0.4–0.5)	0.6 (0.5–0.7)	0.044
Time to THR	M72	M72	NS
** ia-CS–N = 25 **	**N = 10**	**N = 15**	
WOMAC-score	76 (75–77)	78 (76–80)	0.044
Biomarkers-score	400 (350–492)	485 (420–575)	0.041
BMD-score	3.2 (3.0–3.4)	2.8 (2.5 -3.0)	0.442
mJSN	0.5 (0.45–0.55)	0.7 (0.6–0.8)	0.043
Time to THR	M72	M60	0.001
** ia-HA–N = 25 **	**N = 10**	**N = 15**	
WOMAC-score	74 (73–75)	76 (75–77)	0.044
Biomarkers-score	390 (340–440)	440 (390–490)	0.041
BMD-score	3.3 (3.0–3.7)	2.9 (2.6–3.1)	0.442
mJSN	0.45 (0.4–0.5)	0.6 (0.5–0.7)	0.044
Time to THR	M80	M70	0.003
** K/L-III–N = 125 (F75/M50) **	N = 50	N = 75	
** SC–N = 50 **	**N = 20**	**N = 30**	
WOMAC-score	85 (85–86)	87 (86–88)	0.044
Biomarkers-score	490 (440–540)	544 (494–594)	0.041
BMD-score	2.9 (2.7–3.3)	2.4 (2.2–2.6)	0.041
mJSN	0.6 (0.5–0.8)	0.8 (0.7–0.9)	0.042
Time to THR	M54	M42	0.001
** ZA–N = 25 **	**N = 10**	**N = 15**	
WOMAC-score	85 (85–86)	83 (83–84)	0.044
Biomarkers-score	440 (405–475)	480 (440–520)	0.044
BMD-score	3.2 (3.0–3.4)	3.1 (2.9–3.3)	NS
mJSN	0.55 (0.5–0.6)	0.65 (0.6–0.7)	0.044
Time to THR	M54	M54	NS
** ia-CS–N = 25 **	**N = 10**	**N = 15**	
WOMAC-score	85 (85–86)	87 (86–88)	0.044
Biomarkers-score	490 (440–540)	544 (494–594)	0.041
BMD-score	2.9 (2.7–3.3)	2.4 (2.2–2.6)	0.041
mJSN	0.6 (0.5–0.8)	0.8 (0.7–0.9)	0.042
Time to THR	M54	M42	0.001
** ia-HA–N = 25 **	**N = 10**	**N = 15**	
WOMAC-score	83 (83–84)	85 (84–86)	0.044
Biomarkers-score	390 (340–440)	440 (390–490)	0.041
BMD-score	3.3 (3.0–3.7)	2.9 (2.6 -3.1)	0.442
mJSN	0.55 (0.5–0.6)	0.65 (0.6–0.7)	0.044
Time to THR	M60	M50	0.003

Cluster 2#: male-gender associated; Cluster 2*: female-gender associated; WOMAC score: WOMAC-A + WOMAC-C; biomarkers score^:^ levels of s-CTX-I + levels of u-CTX-II; BMD-score: LS-BMD + PF-BMD + TB-BMD); and *p*-value †: Kruskal–Wallis test was applied.

**Table 7:** Multiple linear regression assessment of the changes in radiographic progression (mJSW), according to the type of FHM (b1. Sup.) before and after adjustment for gender (b2. Fem.).

Regression Equation	Adjusted R^2	b1	SD_b1	95% CI_b1	b2	SD_b2	95% CI_b2
**JSW12** = b1. Sup. + b0	0.808	−0.926	0.059	−1.044; −0.809			
**JSW12** = b1. Sup. + b2. Fem. + b0	0.871	−0.926	0.048	−1.023; −0.830	−0.160	0.029	−0.219; −0.101
**JSW24** = b1. Sup. + b0	0.789	−1.113	0.075	−1.263; −0.963			
**JSW24** = b1. Sup. + b2. Fem. + b0	0.911	−1.113	0.048	−1.210; −1.016	−0.267	0.030	−0.327; −0.208
**JSW36** = b1. Sup. + b0	0.695	−1.413	0.121	−1.656; −1.170			
**JSW36** = b1. Sup. + b2. Fem.+ b0	0.892	−1.413	0.072	−1.558; −1.268	−0.457	0.044	−0.546; −0.369

SD: standard deviation; 95% CI: 95% confidence interval; Sup.: superior pattern of FHM; Fem: females.

**Table 8:** Multiple logistic regression of the impact of both types of FHM (superior vs. medial/axial) on radiographic progression, after adjusting for the sex variable.

FHM	OR (95% CI)	*p*-Value	OR (95% CI) *	*p*-Value *
Superior FHM	0.307 (0.103–0.918)	0.031	0.256 (0.076–0.718)	0.024
Medial/axial FHM	0.394 (0.105–0.927)	0.044	0.309 (0.109–0.921)	0.041

*p*-value *: Indicates *p*-values after adjusting for the sex variable.

**Table 9:** Multiple logistic regression of the impact of the two types of FHM and patients’ sex on the radiographic progression in the different clusters.

Variables	Cluster 1		Cluster 2		Cluster 3	
	OR (95% CI)	*p*-Values	OR (95% CI)	*p*-Values	OR (95% CI)	*p*-Values
Sex–F	1.833 (1.012–3.321)	0.045	2.212 (1.182–4.141)	0.013	1.789 (1.034–3.956)	0.045
FHM–superior	1.743 (1.023–3.678)	0.033	2.371 (1.171–4.726)	0.031	1.757 (1.044–3.589)	0.034
FHM–medial	1.761 (1.041–3.658)	0.044	2.387 (1.179–4.232)	0.044	1.821 (1.023–3.331)	0.045

**Table 10:** Treatment effects as assessed by changes in ‘times to THR’ between different clusters and different treatment groups: *p* ^†^—values from the comparisons C1 vs. C2^#^; *p* ^‡^—values from the comparisons C2^#^ vs. C*; *p* ^¥^—values from the comparisons C* vs. C3; P^1^—values from the comparisons SC vs. ia-CS; P^2^—values from the comparisons SC vs. ia-HA; P^3^—values from the comparisons ia-CS vs. ia-HA; P^4^—values from the comparisons SC vs. ZA; P^5^—values from the comparisons ia-CS vs. ZA; and P^6^—values from the comparisons ia-HA vs. ZA. A Kruskal–Wallis test was applied with Holm-Bonferroni post hoc analysis and correction on adjusted *p*-values to limit the bias associated with multiple statistical tests. NA—not applicable.

Time to THR	Cluster 1	*p* ^†^	Cluster 2#	*p* ^‡^	Cluster 2*	*p* ^¥^	Cluster 3
**SC group:**							
K/L-III	M54	**0.034**	M48	**0.034**	M42	**0.044**	M37
K/L-II	M72	**0.034**	M66	**0.034**	M60	**0.034**	M54
**ia-CS group:**							
K/L-III	M54	**0.034**	M48	**0.021**	M40	**NA**	NA
K/L-II	M72	**0.034**	M66	**0.001**	M54	**NA**	NA
P^1^ K/L-II	1.0		1.0		NS	**NA**	NA
P^1^ K/L-III	1.0		1.0		0.034	**NA**	NA
**ia-HA group:**							
K/L-III	M72	**0.034**	M66	**0.001**	M54	**0.001**	M42
K/L-II	M96	**0.034**	M84	**0.001**	M72	**0.001**	M60
P^2^ K/L-II	<0.001		<0.001		0.001		0.044
P^2^ K/L-III	<0.001		<0.001		0.001		0.034
P^3^ K/L-II	<0.001		<0.001		<0.001		NA
P^3^ K/L-III	<0.001		<0.001		<0.001		NA
**ZA group:**							
K/L-III	M54	**1.0**	M54	**1.0**	M54	**0.001**	M42
K/L-II	M72	**1.0**	M72	**1.0**	M72	**0.001**	M66
P^4^ K/L-III	1.0		0.034		0.001		0.044
P^4^ K/L-II	1.0		0.034		0.001		0.001
P^5^ K/L-III	1.0		0.034		<0.001		NA
P^5^ K/L-II	1.0		0.034		<0.001		NA
P^6^ K/L-III	<0.001		0.001		1.0		1.0
P^6^ K/L-II	<0.001		0.001		1.0		0.034

## Data Availability

The primary data used in this study are published and freely available online-author references [31,34]. The source (raw) data used in [31,34] and for this study are available on request from the corresponding author due to the fact that they are owned by the institution where the authors work (University Hospital “Pulmed”—Plovdiv), which hospital is also private, not public or state.

## Supplementary Materials

The following supporting information can be downloaded at: https://www.mdpi.com/article/10.3390/life14050622/s1, Table S1: Descriptive analyses of the 4 composite variables and their components separately for the 3 clusters; Table S2: Descriptive analyses of the four composite variables and their components separately for the M36 clusters; Table S3: Descriptive analyses and comparisons of analgesia with Diclofenac sodium 2 × 75 mg/24 h vs. Tramadol hydrochloride 2 × 100 mg/24 h.

## Abbreviations

WOMAC-A, B, C, T—Western Ontario and McMasters Universities Osteoarthritis index; A;B;C;T—subscales for Pain (A); Stiffness (B); Functional ability (C) and Total (composite index A+B+C); PtGA—Patient Global Assessment; PTH—parathyroid hormone; s-CTX-I—serum β-beta-isomerized carboxy-terminal cross-linking telopeptide of type I collagen; u-CTX-II—urine C-terminal crosslinking telopeptides of collagen type II; mJSW–mean Joint Space Width; JSN mm/yearly—Joint Space Narrowing; DXA—Dual-energy X-ray Absorptiometry; HAL—Hip Axis Length; NSA—Neck Shaft Angle; MNW—Minimal Neck Width; TH-BMD—Total Hip Bone Mineral Density; LS-BMD—Lumbar Spine BMD; TB-BMD–Total Body BMD; MCII—Minimal Clinical Important Improvement; RPs—Radiological Patterns; K/L-II’A’/’I’/’H’—Kellgren-Lawrence grade II with Atrophic/Intermediate (normotrophic)/Hypertrophic Pattern.
