# Supplementary material for: Endophenotypes of Primary Osteoarthritis of the Hip Joint in the Bulgarian Population over 60 Years Old
Source: Life (Basel). 2024 May 11;14(5):622. doi: 10.3390/life14050622 (PMC11122795; doi:10.3390/life14050622)
Supplement: Supplementary file 1 [file life-14-00622-s001.zip › life-2998985-supplementary.pdf]

Table S1 (former table 5). Descriptive analyses of the 4 composite variables and their components separately for the 3 clusters

*	*cluster 1 vs. cluster 2 (p†; p‡)*	*cluster 1 vs. cluster 3 († ; ‡)*	*cluster 2 vs. cluster 3 († ; ‡)*	*P-adj. (1; 2; 3)*
				$P^1=0.044$
WOMAC-scores *K/L-II*	$p^{\dagger}=0.023$	$p^{\dagger}=0.0004$ $p^{\ddagger}=0.0004$	$p^{\dagger}=0.022$	$P^2=0.001$
K/L-III	$p^{\ddagger}=0.022$		$p^{\ddagger}=0.021$	$P^3=0.042$
WOMAC-A				$P^1=0.027$
K/L-II	$p^{\dagger}=0.017$	$p^{\dagger}=0.0002$ $p^{\ddagger}=0.0001$	$p^{\dagger}=0.016$	$P^2=0.003$
K/L-III	$p^{\ddagger}=0.016$		$p^{\ddagger}=0.015$	$P^3=0.025$
WOMAC-C –values				$P^1=0.029$
K/L-II	$p^{\dagger}=0.019$	$p^{\dagger}=0.0003$ $p^{\ddagger}=0.0002$	$p^{\dagger}=0.019$	$P^2=0.004$
K/L-III	$p^{\ddagger}=0.018$		$p^{\ddagger}=0.018$	$P^3=0.027$
Biomarkers scores				$P^1=0.044$
K/L-II	$p^{\dagger}=0.023$	$p^{\dagger}=0.0004$ $p^{\ddagger}=0.0003$	$p^{\dagger}=0.022$	$P^2=0.001$
K/L-III	$p^{\ddagger}=0.022$		$p^{\ddagger}=0.021$	$P^3=0.043$
25-OH-D –values				$P^1=0.021$
K/L-II	$p^{\dagger}=0.007$	$p^{\dagger}=0.00002$ $p^{\ddagger}=0.00001$	$p^{\dagger}=0.006$	$P^2=0.002$
K/L-III	$p^{\ddagger}=0.006$		$p^{\ddagger}=0.005$	$P^3=0.017$
s-CTH-I –values				$P^1=0.027$
K/L-II	$p^{\dagger}=0.022$	$p^{\dagger}=0.003$	$p^{\dagger}=0.017$	$P^2=0.005$
K/L-III	$p^{\ddagger}=0.021$	$p^{\ddagger}=0.002$	$p^{\ddagger}=0.015$	$P^3=0.025$
u-CTX-II –values				$P^1=0.028$
K/L-II	$p^{\dagger}=0.024$	$p^{\dagger}=0.0004$ $p^{\ddagger}=0.0003$	$p^{\dagger}=0.019$	$P^2=0.007$
K/L-III	$p^{\ddagger}=0.022$		$p^{\ddagger}=0.017$	$P^3=0.025$
BMD-scores				$P^1=0.043$
K/L-II	$p^{\dagger}=0.023$	$p^{\dagger}=0.0004$ $p^{\ddagger}=0.0004$	$p^{\dagger}=0.022$	$P^2=0.008$
K/L-III	$p^{\ddagger}=0.021$		$p^{\ddagger}=0.021$	$P^3=0.042$
TB-BMD				$P^1=0.026$
K/L-II	$p^{\dagger}=0.019$	$p^{\dagger}=0.0002$ $p^{\ddagger}=0.0001$	$p^{\dagger}=0.016$	$P^2=0.003$
K/L-III	$p^{\ddagger}=0.017$		$p^{\ddagger}=0.015$	$P^3=0.025$
LS-BMD				$P^1=0.027$
K/L-II	$p^{\dagger}=0.016$	$p^{\dagger}=0.0002$ $p^{\ddagger}=0.0001$	$p^{\dagger}=0.016$	$P^2=0.002$
K/L-III	$p^{\ddagger}=0.015$		$p^{\ddagger}=0.015$	$P^3=0.025$
PF-BMD				$P^1=0.029$
K/L-II	$p^{\dagger}=0.021$	$p^{\dagger}=0.0004$ $p^{\ddagger}=0.0003$	$p^{\dagger}=0.019$	$P^2=0.004$
K/L-III	$p^{\ddagger}=0.019$		$p^{\ddagger}=0.018$	$P^3=0.027$
mJSW				$P^1=0.033$
K/L-II	$p^{\dagger}=0.017$	$p^{\dagger}=0.0003$ $p^{\ddagger}=0.0004$	$p^{\dagger}=0.015$	$P^2=0.005$
K/L-III	$p^{\ddagger}=0.018$		$p^{\ddagger}=0.016$	$P^3=0.034$

WOMAC score: WOMAC-A + WOMAC-C; Biomarker scores: levels of s-CTX-I + levels of u-CTX-II; BMD score: LS-BMD + PF-BMD + TB-BMD; *: indicates the use of the Kruskal-Wallis test; †: indicates the p-values of comparisons in the K/L-II grade; ‡: indicates the p-values of comparisons in the K/L-III grade; p adj.: adjusted p-values using the Holm-Bonferroni post hoc correction to limit the bias associated with multiple statistical tests: P^1: indicates p-values of the comparisons between cluster-1 and cluster-2; P^2: indicates the p-values of the comparisons between cluster-1 and cluster-3; P^3: indicates p-values of the comparisons between cluster-2 and cluster-3.

Table S2 (former table 8). Descriptive analyses of the four composite variables and their components separately for the M36 clusters

*	*cluster 1 vs. cluster 2*$^{\#}$ *(*$p^{\dagger}$*;* $p^{\ddagger}$*)*	*cluster 1 vs. cluster 3 (*†*;* ‡*)*	*cluster 2* vs. cluster 3 (*†*;* ‡*)*	*P-adj. (*^{1}*;* ^{2}*;* ^{3}*)*
WOMAC-scores				P^1=0.044
K/L-II	$p^{\dagger}$=0.023	$p^{\dagger}$=0.0004	$p^{\dagger}$=0.023	P^2=0.001
K/L-III	$p^{\ddagger}$=0.022	$p^{\ddagger}$=0.0004	$p^{\ddagger}$=0.022	P^3=0.044
WOMAC-A –values				P^1=0.033
K/L-II	$p^{\dagger}$=0.022	$p^{\dagger}$=0.0002	$p^{\dagger}$=0.017	P^2=0.003
K/L-III	$p^{\ddagger}$=0.021	$p^{\ddagger}$=0.0001	$p^{\ddagger}$=0.016	P^3=0.033
WOMAC-C –values				P^1=0.033
K/L-II	$p^{\dagger}$=0.019	$p^{\dagger}$=0.0003	$p^{\dagger}$=0.019	P^2=0.004
K/L-III	$p^{\ddagger}$=0.018	$p^{\ddagger}$=0.0002	$p^{\ddagger}$=0.018	P^3=0.033
Biomarkers scores	$p^{\dagger}$=0.024			P^1=0.044
K/L-II	$p^{\dagger}$=0.022	$p^{\dagger}$=0.0004	$p^{\dagger}$=0.023	P^2=0.001
K/L-III	$p^{\ddagger}$=0.021	$p^{\ddagger}$=0.0003	$p^{\ddagger}$=0.022	P^3=0.043
s-CTH-I –values				P^1=0.032
K/L-II	$p^{\dagger}$=0.022	$p^{\dagger}$=0.003	$p^{\dagger}$=0.023	P^2=0.005
K/L-III	$p^{\ddagger}$=0.021	$p^{\ddagger}$=0.002	$p^{\ddagger}$=0.021	P^3=0.031
u-CTX-II –values				P^1=0.028
K/L-II	$p^{\dagger}$=0.023	$p^{\dagger}$=0.0004	$p^{\dagger}$=0.024	P^2=0.007
K/L-III	$p^{\ddagger}$=0.022	$p^{\ddagger}$=0.0003	$p^{\ddagger}$=0.022	P^3=0.029
BMD-scores				P^1=0.043
K/L-II	$p^{\dagger}$=0.023	$p^{\dagger}$=0.0004	$p^{\dagger}$=0.024	P^2=0.008
K/L-III	$p^{\ddagger}$=0.021	$p^{\ddagger}$=0.0004	$p^{\ddagger}$=0.022	P^3=0.042
TB-BMD				P^1=0.026
K/L-II	$p^{\dagger}$=0.019	$p^{\dagger}$=0.0002	$p^{\dagger}$=0.019	P^2=0.003
K/L-III	$p^{\ddagger}$=0.017	$p^{\ddagger}$=0.0001	$p^{\ddagger}$=0.017	P^3=0.025
LS-BMD				P^1=0.027
K/L-II	$p^{\dagger}$=0.016	$p^{\dagger}$=0.0002	$p^{\dagger}$=0.016	P^2=0.002
K/L-III	$p^{\ddagger}$=0.015	$p^{\ddagger}$=0.0001	$p^{\ddagger}$=0.015	P^3=0.026
PF-BMD				P^1=0.029
K/L-II	$p^{\dagger}$=0.021	$p^{\dagger}$=0.0004	$p^{\dagger}$=0.021	P^2=0.004
K/L-III	$p^{\ddagger}$=0.019	$p^{\ddagger}$=0.0003	$p^{\ddagger}$=0.019	P^3=0.027
mJSN				P^1=0.043
K/L-II	$p^{\dagger}$=0.017	$p^{\dagger}$=0.0003	$p^{\dagger}$=0.017	P^2=0.005
K/L-III	$p^{\ddagger}$=0.018	$p^{\ddagger}$=0.0004	$p^{\ddagger}$=0.018	P^3=0.044

WOMAC-score: WOMAC-A + WOMAC-C; Biomarker score: levels of s-CTX-I + levels of u-CTX-II; BMD-score: LS-BMD + PF-BMD + TB-BMD;*: indicates that the Kruskal–Wallis test was applied; †: indicates p-values of the comparisons in the K/L-II grade; ‡: indicates p-values of the comparisons in the K/L-III grade; P-adj.: adjusted p-values using the Holm-Bonferroni post hoc correction to limit the bias associated with multiple statistical tests): P^1: p-values of the comparisons between cluster 1 and cluster 2$^{\#}$; P^2: p-values of the comparisons between cluster 1 and cluster 3; P^3: p-values of the comparisons between cluster 2* and cluster 3.

Table S3. Descriptive analyses and comparisons of analgesia with Diclofenac sodium 2x75mg/24h vs. Tramadol hydrochloride 2x100mg/24h

*	p-values† Cluster 1	p-values† Cluster 2#	p-values† Cluster 2*	p-values† Cluster 3	P-adj. (1; 2; 3; 4; 5)
WOMAC-scores	p† = 0.079	p† = 0.059	p† = 0.021	p† = 0.0001	P1 = 0.045<br>P2 = 0.023<br>P3 = 0.001<br>P4 = 0.026<br>P5 = 0.042

WOMAC score: WOMAC-A + WOMAC-C; *: indicates that the Kruskal–Wallis test was applied; †: indicates p - values of the comparisons Diclofenac sodium 2x75mg/24h vs. Tramadol hydrochloride 2x100mg/24h; P-adj.: adjusted p-values using the Holm-Bonferroni post hoc correction to limit the bias associated with multiple statistical tests): P1: p-values of the comparisons of p†, between cluster 1 and cluster 2#; P2: p-values of the comparisons of p†, between cluster 1 and cluster 2*; P3: p-values of the comparisons of p†, between cluster 1 and 3; P4: p-values of the comparisons of p†, between cluster 2# and 2*; P5: p-values of the comparisons of p†, between cluster 2* and 3.
